# Distinct Transcriptional and Alternative Splicing Signatures of Decidual CD4^+^ T Cells in Early Human Pregnancy

**DOI:** 10.3389/fimmu.2017.00682

**Published:** 2017-06-12

**Authors:** Weihong Zeng, Zhicui Liu, Xinmei Liu, Siming Zhang, Asma Khanniche, Ying Zheng, Xiaoling Ma, Tiantian Yu, Fuju Tian, Xiao-Rui Liu, Jianxia Fan, Yi Lin

**Affiliations:** ^1^Institute of Embryo-Fetal Original Adult Disease Affiliated to Shanghai Jiao Tong University School of Medicine, The International Peace Maternity & Child Health Hospital, Shanghai Jiao Tong University School of Medicine, Shanghai, China; ^2^Department of Dermatology, Ruijin Hospital, Shanghai Jiaotong University School of Medicine, Shanghai, China; ^3^Shanghai Institute of Immunology, Shanghai Jiao Tong University School of Medicine, Shanghai, China; ^4^Out-patient Operating Room, The International Peace Maternity and Child Health Hospital, Shanghai Jiao Tong University School of Medicine, Shanghai, China

**Keywords:** decidual CD4^+^ T cells, early human pregnancy, transcriptome, alternative splicing, high-throughput mRNA sequencing

## Abstract

Decidual CD4^+^ T (dCD4 T) cells are crucial for the maternal-fetal immune tolerance required for a healthy pregnancy outcome. However, their molecular and functional characteristics are not well elucidated. In this study, we performed the first analysis of transcriptional and alternative splicing (AS) landscapes for paired decidual and peripheral blood CD4^+^ T (pCD4 T) cells in human early pregnancy using high throughput mRNA sequencing. Our data showed that dCD4 T cells are endowed with a unique transcriptional signature when compared to pCD4 T cells: dCD4 T cells upregulate 1,695 genes enriched in immune system process whereas downregulate 1,011 genes mainly related to mRNA catabolic process and the ribosome. Moreover, dCD4 T cells were observed to be at M phase, and show increased activation, proliferation, and cytokine production, as well as display an effector-memory phenotype and a heterogenous nature containing Th1, Th17, and Treg cell subsets. However, dCD4 T cells undergo a comparable number of upregulated and downregulated AS events, both of which are enriched in the genes related to cellular metabolic process. And the changes at the AS event level do not reflect measurable differences at the gene expression level in dCD4 T cells. Collectively, our findings provide a comprehensive portrait of the unique transcriptional signature and AS profile of CD4^+^ T cells in human decidua and help us gain more understanding of the functional characteristic of these cells during early pregnancy.

## Introduction

The maternal-fetal interface (MFI) is regarded as the interface between the extraembryonic tissues of developing conceptus and the uterine mucosa, and the immune cells at the MFI are the maternal immune cells that populate the decidua (1–3). During the first trimester of healthy pregnancy, natural killer (NK) cells, macrophages, and T cells are the most abundant leukocytes in human decidua, whereas dendritic cells (DCs), B cells and NKT cells are rare (3). Since these decidual leukocytes are reported to play a key role in facilitating tolerance to the semi-allogeneic fetus and therefore maintaining a successful pregnancy, a deeper understanding of the molecular and functional features of these cells will provide novel insights into the pathogenesis of pregnancy-related complications and poor postnatal health (3–5). In first-trimester human decidua, approximately 70% of leukocytes are decidual NK (dNK) cells, which display a phenotype of CD56^bright^CD16^dim^ and play important roles in promoting neo-angiognesis and trophoblast invasion, remodeling of spiral arteries, and directing placentation (6–9). Whole-genome transcriptome analysis revealed that dNK cells upregulate expression of many genes related to inhibitory receptors, growth factors, cell cycle, and cytokines/chemokines, but downregulate expression of the genes involved in activating receptors and costimulatory factors as compared with peripheral blood NK cells (10, 11).

Unlike dNK cells, decidual T cells are less abundant and do not have a trophic function. However, they are thought to play rather important roles in immune regulation and allograft tolerance at the MFI (3, 12, 13). In early human pregnancy, about 10–20% of decidual leukocytes are CD3^+^ TCRαβ^+^ T cells, and approximately 30–45% of them are CD4^+^ T cells, that display an activated/memory CD25^dim^ cell surface phenotype (3, 14–16). More than 20 years ago, Saito and colleagues observed that in the first trimester of human normal pregnancy, decidual CD4^+^ T (dCD4 T) cells express the T-cell-activation antigens CD69, HLA-DR, interleukin-2 receptor-alpha (IL-2Rα), and IL-2Rβ at a significantly higher level than do peripheral blood CD4^+^ T (pCD4 T) cells, indicating that dCD4 T cells are regionally activated at an early stage of pregnancy (17). Furthermore, recent studies showed that dCD4 T cells in early pregnancy express higher levels of the T regulatory (Treg)-cell markers CD25 and FOXP3, the proliferation-associated antigen Ki-67, programmed cell death-1 (PDCD1, also called PD-1), and T-cell immunoglobulin mucin-3 (Tim-3) than their peripheral blood counterparts (13, 18–20). However, the complexity of dCD4 T cells has not been well elucidated, and a thorough understanding of the molecular and functional features of these cells would shed light on their eventual roles during early pregnancy.

Upon encountering antigens presented by antigen-presenting cells (APCs) and being driven by a set of transcriptional regulators and cytokines, naive CD4^+^ T helper (Th) cells are able to differentiate into distinct effector subsets, including Th1, Th2, Th17, and Treg cells (5, 21, 22). Th1, Th2, and Th17 cells are characterized by their production of IFN-γ, IL-4, and IL-17, respectively. Their differentiation is controlled by the following lineage-specific “master” transcription factors: T-box-binding transcription factor (T-bet) for Th1, GATA-binding protein 3 (GATA3) for Th2, and RAR-related orphan receptor gamma (RORγ) for Th17 cells; whereas Treg cells are defined and driven by expressing the forkhead transcription factor FOXP3 (5, 13, 21, 22). Although growing evidences suggest that Treg cells in the decidua contribute to maternal-fetal immune tolerance and pregnancy maintenance, the role and necessity of other decidual Th-cell subsets in a normal pregnancy is still largely unknown or remains controversial (3, 16, 23, 24).

In eukaryotic organisms, alternative splicing (AS) is a crucial and ubiquitous mechanism that regulates gene expression and generates transcript/protein diversity by producing two or more distinct mRNA transcripts from the same precursor mRNA (pre-mRNA) using different splice sites (25, 26). Nearly 75% of all human genes and 94% of human multiexonic genes undergo AS, with a bias toward genes expressed in the nervous and immune systems, and toward developmental stage-specific and tissue-specific expression (27–29). Moreover, AS plays a crucial role in shaping the T-cell response to stimulation (30), and in the process of CD4^+^ T-cell activation (31) and differentiation (32). In CD4^+^ T cells, many genes with known functions in immunobiology have been reported to undergo AS changes during immune responses, including genes coding for cell surface and adapter proteins (e.g., *CD45, CD96, CTLA-4*), transcriptional regulators (e.g., *GATA3, FOXP3, HIF1*α), RNA-processing (e.g., *AUF-1, CELF2, EIF4G2*) and intracellular signaling/transport molecules (e.g., *AKAP9, CLK2, MAP3K7*), and cytokines (e.g., *IL4, TIR8*) (25, 30, 33, 34). As yet, little is known about the genome-wide AS profile of CD4^+^ T cells in human decidua as compared to those in the peripheral blood.

In this study, we compared the transcriptional profile and AS landscape between paired decidual and peripheral CD4^+^ T cells from healthy women at the first trimester of pregnancy using high-throughput mRNA sequencing (mRNA-Seq), which can generate quantitative measurements of gene expression and AS events, as well as provide greater accuracy and sensitivity than microarrays (35, 36). Our findings are the first to show that dCD4 T cells upregulate genes related to immune system process and downregulate genes involved in mRNA catabolic process, whereas AS is not a major contributor to these changes. Our results thus provide new insights into the molecular traits of CD4^+^ T cells in human decidua during early pregnancy.

## Materials and Methods

### Subjects

This study was approved and performed in compliance with the Medical Ethics Committee of the International Peace Maternity and Child Health Hospital of China Welfare Institute (Shanghai, China). Twelve healthy pregnant women with no history of spontaneous abortion, preterm labor or preeclampsia in their previous pregnancies were recruited for this study. Their blood samples and decidual tissues were obtained while they were undergoing elective surgical abortion at the department of Obstetrics and Gynecology in the International Peace Maternity and Child Health Hospital of China Welfare Institute affiliated to Shanghai Jiao Tong University School of Medicine. Informed consent was obtained from all participants in written form before enrollment. Venous blood was collected in EDTA-anticoagulant tubes (BD Vacutainer, USA) immediately before termination of pregnancy, and the autologous decidual tissues were collected and stored in sterile ice-cold phosphate-buffered saline (PBS). Samples from three pregnant women (mean age, 26 years; range, 22–28 years) with mean Gestational Day 50 (range, Days 44–58) were used for mRNA-Seq analysis; those from other five women (mean age, 30 years; range, 22–39 years) with mean Gestational Day 45 (range, Days 38–50) were used for validation of the mRNA-Seq data by flow cytometry staining, whereas samples from the last four women (mean age 34 years, range 30–39; mean Gestational Day 45, range 43–50) were applied for determination of CD4^+^ T-cell functional status (Figure S1 in Supplementary Material).

### Separation of Mononuclear Cells

Separation of decidual mononuclear cells (DMCs) from decidual tissues was performed according to the procedure of non-enzymatic leukocytes separation, as described previously (5, 13, 37–39). Briefly, vacuum-aspirated abortion tissues were washed in sterile PBS; the decidual tissue was separated macroscopically from the fetal tissue and placenta, and then cut into small pieces (<1 mm^3^) using ocular scissors and filtered through a 74-µm nylon mesh filter to obtain a single cell suspension. Both DMCs and peripheral blood mononuclear cells (PBMCs) were isolated and purified by density gradient centrifugation using Lymphoprep™ (AS1114546, Axis-shield).

### Monoclonal Antibodies (mAbs) and Reagents

Fluorescein-conjugated anti-human mAbs, including anti-CD3 FITC (clone: UCHT1), anti-CD4 V500 (RPA-T4), anti-CD8 APC/Cy7 (SK1), anti-CD56 PE (B159), and anti-CD279 (PD-1) PE (EH12.1, also known as EH12) were purchased from BD Pharmingen. Meanwhile, the other anti-human mAbs including anti-CD3 APC (HIT3a), anti-CD8a PerCP/Cy5.5 (HIT8a), anti-CD38 FITC (HIT2), anti-CD63 FITC (H5C6), anti-CD69 APC (FN50), anti-CD122 (IL-2Rβ) APC (TU27), anti-CD183 (CXCR3) PE/Cy7 (G025H7), anti-CD183 (CXCR3) APC (G025H7), anti-CD192 (CCR2) APC/Cy7 (K036C2), anti-CD196 (CCR6) PE (G034E3), anti-CD197 (CCR7) PE (G043H7), anti-CD276 (B7-H3) PE (DCN.70), anti-CD45RO FITC (UCHL1), anti-CD197 (CCR7) PE/Cy7 (G043H7), anti-IFN-γ APC/Cy7 (4S.B3), anti-IL-4 PE/Cy7 (MP4-25D2), anti-IL-17A FITC (BL168), and anti-FOXP3 PE (206D) were purchased from BioLegend.

### Flow Cytometry

Cell surface and intracellular staining were performed as previously described (5, 40–42). Surface staining of the isolated mononuclear cells (PBMCs and DMCs) was realized by incubating the cells directly with different cocktails of anti-human mAbs in 100 µL PBS containing 3% (v/v) fetal bovine serum at room temperature for 30 min. For intracellular detection of IFN-γ, IL-17A, IL-4, and Foxp3, the isolated mononuclear cells were stimulated with 81 nM phorbol-12-myristate-13-acetate (PMA) and 1.34 µM ionomycin for 4 h in the presence of brefeldin (10.6 µM) and monensin (2 µM) (eBiosciences, USA); cells were stained first for surface markers and then for intracellular cytokines (IFN-γ, IL-17A, IL-4) and Foxp3 by using Foxp3 staining buffers (eBioscience, USA) following the manufacturer’s recommendation. The immunostained cells were collected and analyzed on a BD FACS Canto II flow cytometer (BD Biosciences, USA), and data were processed using the FlowJo 7.6.1 software.

### Purification and RNA Isolation of CD4^+^ T Cells

The decidual and peripheral blood CD4^+^ (dCD4 and pCD4) T cells were purified from the DMCs and PBMCs, respectively, by sorting on a FACS Aria II (BD Biosciences, USA) based on the surface expression of CD56, CD3, and CD4 markers (CD56^−^CD3^+^CD4^+^), always achieving a purity greater than 95%. Total RNA of freshly sorted CD4^+^ T cells was extracted using the Trizol Reagent (Invitrogen, USA) and was purified by RNeasy Micro Kit (Cat No.: 74004, Qiagen, Germany). RNA purity and concentration were determined using a NanoPhotometer^®^ spectrophotometer (IMPLEN, USA) and Qubit^®^ RNA Assay Kit in Qubit^®^ 2.0 Flurometer (Life Technologies, USA), respectively. The RNA integrity was evaluated by the RNA Nano 6000 Assay Kit of the Agilent Bioanalyzer 2100 system (Agilent Technologies, USA) (43).

### Library Preparation, Clustering, and mRNA-Seq

Library preparation, clustering, and mRNA-seq were performed as previously described (43). We used 200 ng of RNA per sample (three samples of both pCD4 and dCD4 T cells) as input material for the RNA sample preparations. cDNA libraries were constructed using the NEBNext^®^ Ultra™ RNA Library Prep Kit for Illumina^®^ (NEB, USA), according to the manufacturer’s instructions. The products were purified using the AMPure XP system and library quality was determined on the Agilent Bioanalyzer 2100 system. We carried out clustering of the index-coded samples on a cBot Cluster Generation System using a HiSeq 2500 PE Cluster Kit (Illumia, USA), following the manufacturer’s recommendations. After cluster generation, the prepared libraries were sequenced on an Illumina Hiseq 2500 platform and 125 bp paired-end reads were produced.

### Sequence Alignment and Gene Expression Analysis

Sequenced reads were aligned to the human reference genome (hg19 version) using the STAR software package (44). Exons from all isoforms of a gene were merged to create one meta-gene. The number of reads falling in the exons of this meta-gene was counted using HTSeq-count, and differential expression analysis was performed using DESeq (45). Differences in gene expression with a *P*-value < 0.05 (paired test) were considered significant. The complete data have been deposited in NCBI Gene Expression Omnibus with accession number GSE97395.

### AS Analysis

rMATS (v3.2.1 beta) was used to identify and assess differential expression of AS events (46). We used the hg19 genome as our reference genome, and transcripts annotation from the NCBI Reference Sequence (47) for AS events annotation. The “exon inclusion level” (Ψ) value was estimated by the percentage of the density of exon inclusion reads among the sum of the densities of exon inclusion reads plus exon skipping reads, as described previously (46, 48). Differential AS events were accepted if they could achieve the threshold of |ΔΨ| > 0.05 and a false discovery rate (FDR) of < 0.05, which were divided into two sets: upregulated AS sets were those with an FDR < 0.05 and a ΔΨ > 0.05, and downregulated AS sets were those with an FDR < 0.05 and a ΔΨ < −0.05.

### Heatmap, Gene Ontology Annotation, KEGG Pathway, and Gene Set Enrichment Analysis (GSEA)

Heatmap analysis of differential genes was performed using R. Functional annotations, including Gene Ontology (GO) annotation and Kyoto Encyclopedia of Genes and Genomes (KEGG) pathway enrichment analysis of interesting genes, were carried out using the DAVID Bioinformatics Resources (http://david.ncifcrf.gov/home.jsp). *P-*values < 0.05 were considered as the significance threshold. GSEA was performed by using the GSEA-P software, MSigDB 1.0, as described previously (49).

## Results

### Distinct Transcriptional Signatures between Human dCD4 and pCD4 T Cells

**Figure 1:**
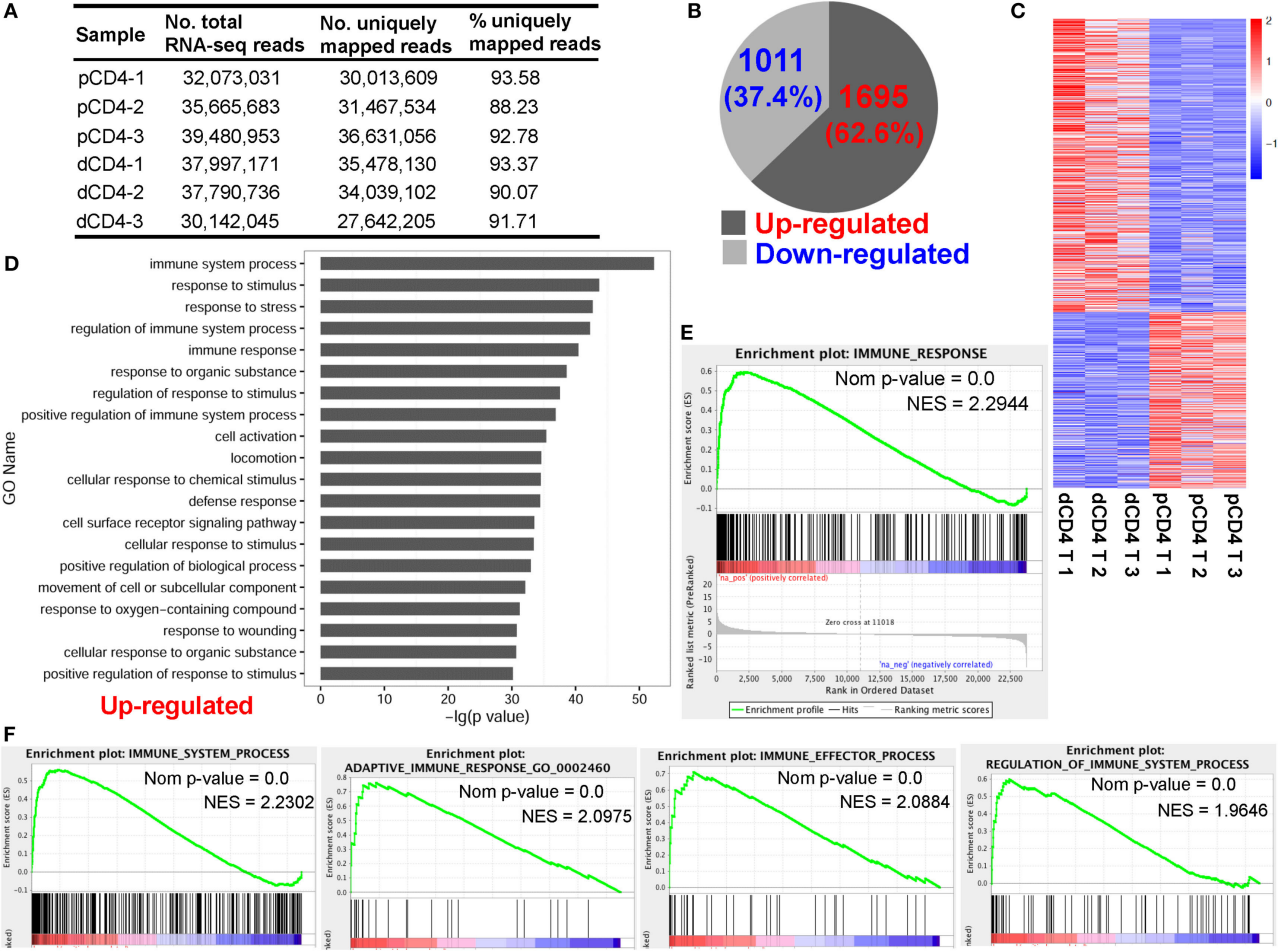
Human dCD4 T cells show a distinct transcriptional signature and upregulate genes related to immune system process as compared with autologous pCD4 T cells. Three healthy women at the first trimester of normal pregnancy were recruited and their dCD4 and pCD4 T cells were isolated by fluorescence-activated cell sorting (FACS). **(A)** Summary of mRNA-Seq data for the purified dCD4 and pCD4 T cells. RNA samples of paired dCD4 and pCD4 T cells from three individuals were sequenced on the Illumina Hiseq 2500 platform, yielding approximately 30–40 million 2 × 125-bp paired-end reads per sample, which were then mapped to the human reference genome (hg19 version). **(B)** Number and percentage of the differentially expressed genes (*P* < 0.05, paired test) that were upregulated or downregulated in dCD4 T cells versus their pCD4 T counterparts. **(C)** Heatmap of differentially expressed genes. Each line represents one gene. Each column represents one sample. Different colors represent the expression levels (from blue to red: increased expression). **(D)** Functional enrichment of the upregulated genes in dCD4 T cells by GO annotation of biological process. The top 20 GO terms are listed. **(E,F)** GSEA plots of GO categories including immune response **(E)**, immune system process, adaptive immune response, immune effector process, and regulation of immune system process **(F)** in dCD4 versus pCD4 T cells. dCD4 T, decidual CD4^+^ T; pCD4 T, peripheral blood CD4^+^ T; No., number; %, percentage; GO, Gene Ontology; GSEA, gene set enrichment analysis; Nom, Nominal; NES, Normalized Enrichment Score.

To elicit the genome-wide transcriptional and AS differences between pCD4 and dCD4 T cells, three healthy women at the first trimester of normal pregnancy were recruited, and fluorescence-activated cell sorting (FACS) was performed to isolate and purify CD4^+^ T cells (CD56^−^CD3^+^CD4^+^) from the decidua and autologous peripheral blood (Figures S1 and S2 in Supplementary Material). Polyadenylated [poly(A)+] RNA samples of both cell populations from each donors were sequenced using Illumina Hiseq 2500 platform, which yielded a total of 213 million 2 × 125-bp paired-end reads, ranging from 30 to 40 million reads per sample (Figure 1A). After aligning all sequenced reads to the human reference genome, between 88 and 94% of reads per sample were mapped uniquely on the hg19 reference (Figure 1A). Multi-loci mapping and unmapped reads were excluded from further analyses.

For mRNA-Seq datasets, the read count is demonstrated to be linearly related and represent a good approximation to the abundance of the target gene (45, 50). In the present study, the number of reads was counted using HTSeq-count and differential expression analysis was performed using DESeq (45). Data showed that there were 2,706 differentially expressed genes between dCD4 and pCD4 T cells, with most of them (62.6%, 1,695 genes) being upregulated and the remaining 1,011 genes (37.4%) being downregulated in dCD4 T cells as compared with autologous pCD4 T cells (Figures 1B,C; Table S1 in Supplementary Material).

### dCD4 T Cells Upregulate the Molecules Related to Immune System Process

Functional enrichment of the upregulated genes in dCD4 T cells by GO annotation of Biological Process (BP) revealed that these genes are most significantly enriched in the category of immune system process (*P* = 4.97E−53), followed by response to stimulus/stress, regulation of immune system process, immune response, regulation of response to stimulus, positive regulation of immune system process, cell activation and the defense response categories (Figure 1D). GSEA has been used widely as a knowledge-based approach to interpret genome-wide expression profiles (49). GSEA using GO categories of BP showed that the gene set of immune response was most positively enriched in dCD4 versus pCD4 T cells (Normalized Enrichment Score = 2.2944), followed by immune system process, defense response, response to external stimulus, adaptive immune response, immune effector process, regulation of immune system process, and inflammatory response (Figures 1E,F; Figure S3 in Supplementary Material). In addition, KEGG pathway and GSEA analyses revealed that the upregulated genes in dCD4 T cells were dramatically enriched in the terms related to antigen processing and presentation (*P* = 2.06E−15 with KEGG analysis; NES = 2.6970 with GSEA), allograft rejection, lysosome, cytokine, and cytokine receptor interaction, Toll-like receptor signaling pathway, and p53 and MAPK signaling pathways (Figure S4 in Supplementary Material).

**Figure 2:**
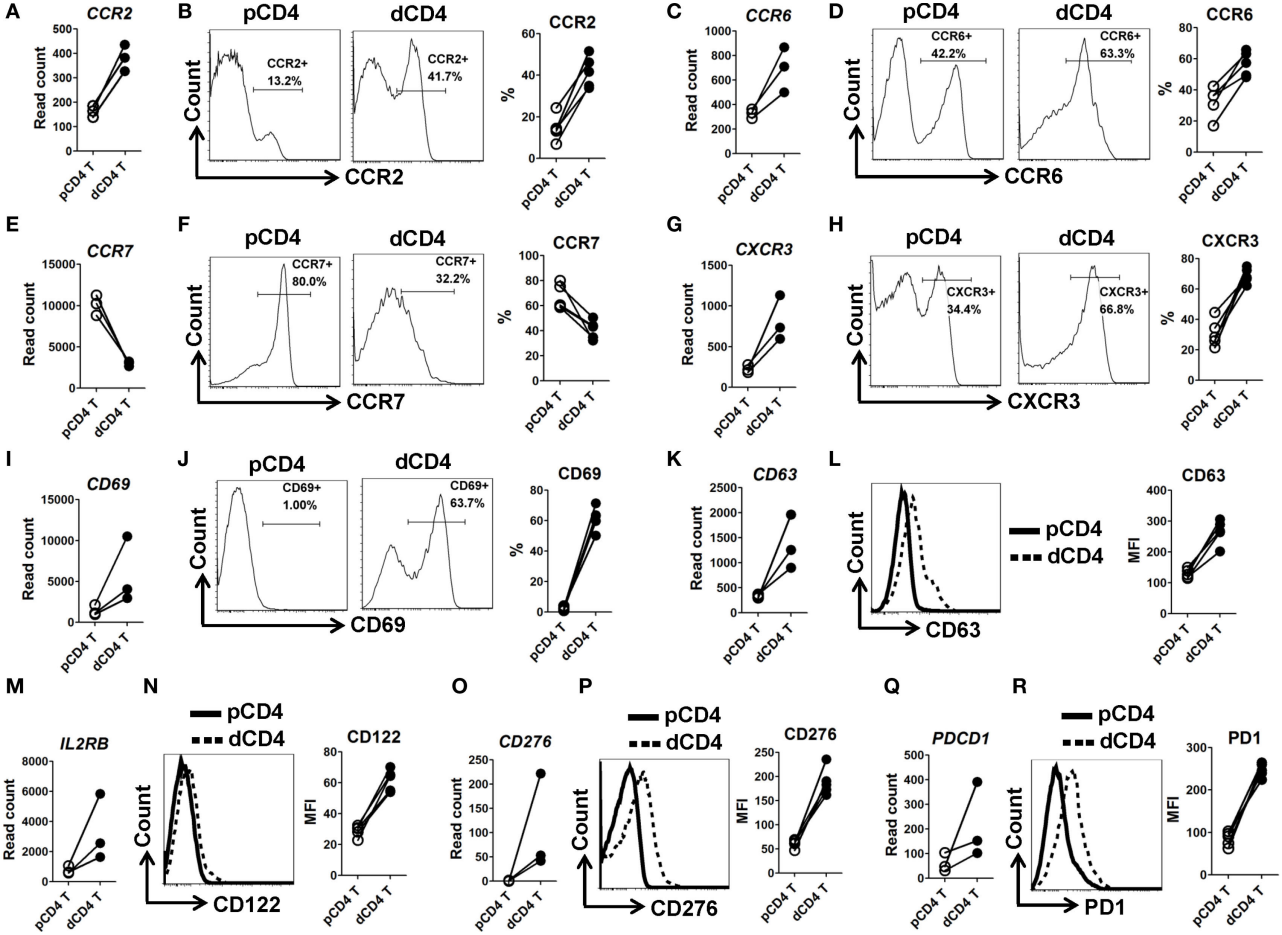
Validation of mRNA-Seq data by flow cytometry staining. **(A,C,E,G,I,K,M,O,Q)** Comparison of the gene expression (measured as the read count) of *CCR2, CCR6, CCR7, CXCR3, CD69, CD63, IL2RB, CD276*, and *PDCD1* between paired pCD4 and dCD4 T cells. Each symbol reflects a sample and each line reflects samples from the same individual (*n* = 3 per group). **(B,D,F,H,J,L,N,P,R)** Representative flow cytometric histograms (left) and cumulative data (right) illustrating the comparison of molecular expression of indicated proteins between paired pCD4 and dCD4 T cells (*n* = 5 per group). The cells were gated in CD4^+^ T cells and the geometric MFI values were calculated using FlowJo 7.6.1 software. pCD4 T, peripheral blood CD4^+^ T; dCD4 T, decidual CD4^+^ T; %, percentage; MFI, mean fluorescent intensity.

In order to validate the mRNA-Seq data, we selected 10 immune response associated molecules and performed flow cytometry staining in five other human samples at the first trimester of normal pregnancy (Figure S1 in Supplementary Material). Being almost completely consistent with the mRNA-Seq data, we observed that human dCD4 T cells highly upregulate CCR2, CCR6, CXCR3, CD38, CD69, CD63, CD122 (encoded by *IL2RB*), CD276, and PD-1 (encoded by *PDCD1*), but downregulate CCR7, as compared with autologous pCD4 T cells (Figure 2; Figure S5 in Supplementary Material).

Taken together, these data showed that human dCD4 T cells upregulate genes mainly related to immune system process, suggesting that these genes might underline an enhanced immune function of dCD4 T cells during human early pregnancy.

### dCD4 T Cells Downregulate Genes Related to mRNA Catabolic Process and the Ribosome

**Figure 3:**
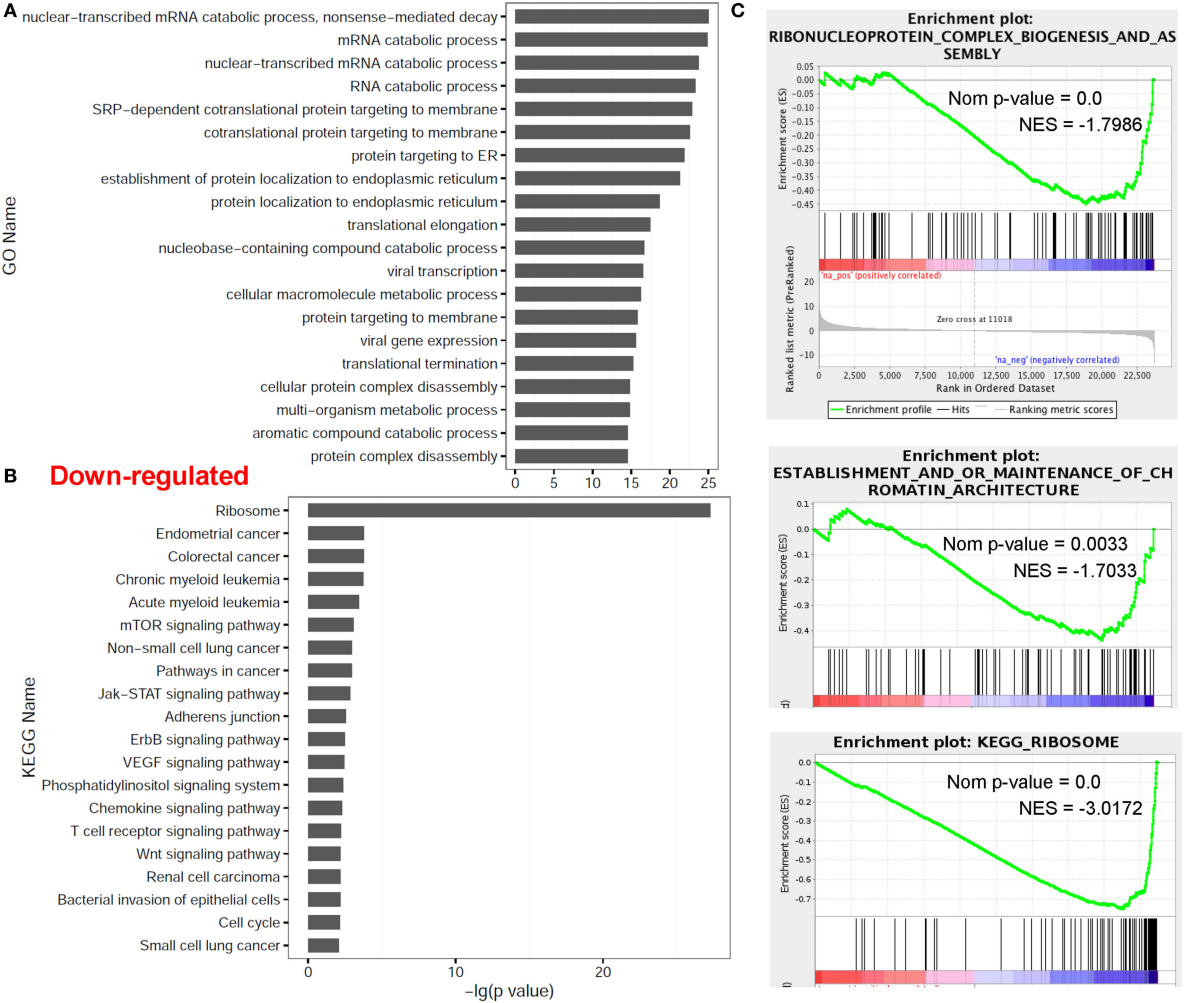
Functional enrichment analyses for genes downregulated in dCD4 versus pCD4 T cells. **(A,B)** The top 20 GO and KEGG terms enriched for downregulated genes in dCD4 versus pCD4 T cells. **(C)** GSEA plots of GO/KEGG categories including ribonucleoprotein complex biogenesis and assembly, establishment and/or maintenance of chromatin architecture, and ribosome. GO, Gene Ontology; KEGG, Kyoto Encyclopedia of Genes and Genomes; GSEA, gene set enrichment analysis; Nom, Nominal; NES, Normalized Enrichment Score.

In contrast, multiple other categories including nuclear-transcribed mRNA catabolic process (*P* = 9.00E−26 with GO annotation), mRNA catabolic process (GO *P* = 1.41E−25), nuclear-transcribed mRNA catabolic process (GO *P* = 1.85E−24), protein RNA complex assembly (NES = −1.81 with GSEA), ribonucleoprotein complex biogenesis and assembly (GSEA NES = −1.77), establishment and/or maintenance of chromatin architecture (GSEA NES = −1.72) and the ribosome (*P* = 5.22E−28 with KEGG analysis; GSEA NES = −3.05), were significantly enriched in the downregulated genes in dCD4 T versus pCD4 T cells by GO annotation, KEGG pathway and/or GSEA analyses (Figure 3). These results indicated that human dCD4 T cells mainly downregulate the genes related to mRNA catabolic process and the ribosome.

### Enhanced Functionality and Subset Complexity of dCD4 T Cells

**Figure 4:**
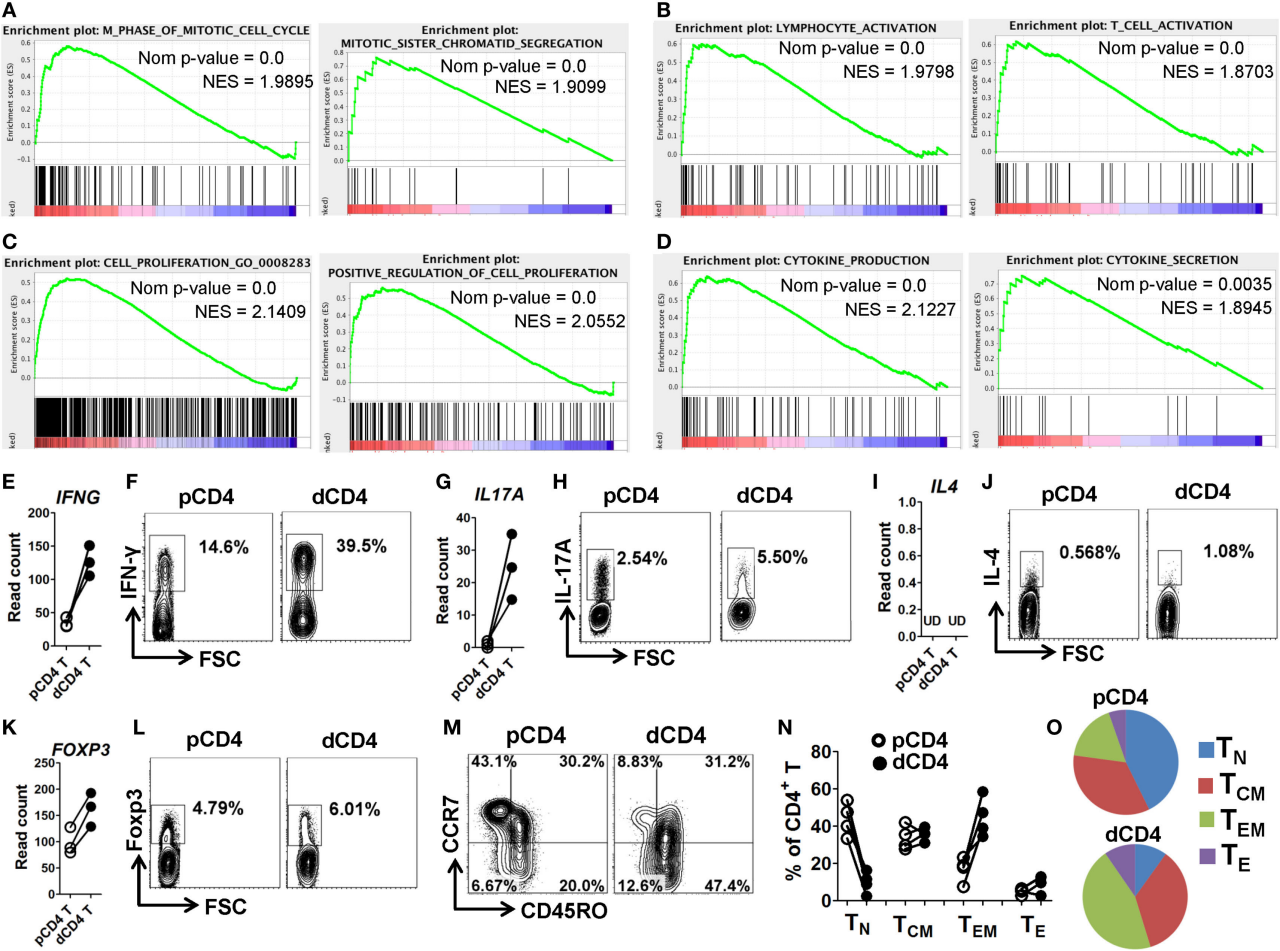
dCD4 T cells stay in M phase and show increased activation, proliferation, and cytokine production, as well as contain Th1, Th17, and Treg cell subsets and display an effector-memory phenotype. **(A–D)** GSEA plots of GO categories, including M phase of mitotic cell cycle, mitotic sister chromatin segregation **(A)**; lymphocyte activation, T cell activation **(B)**; cell proliferation, positive regulation of cell proliferation **(C)**; and cytokine production and cytokine secretion **(D)**. **(E,G,I,L)** Comparison of the gene expression (measured as the read count) of *IFNG, IL17A, IL4*, and *FOXP3* between in paired pCD4 and dCD4 T cells at rest. Each symbol reflects a sample and each line reflects the samples from an individual (*n* = 3 per group). **(F,H,J,K)** Comparison of the expression of IFN-γ, IL-17A, IL-4, and Foxp3 between paired pCD4 and dCD4 T cells as determined by intracellular staining upon stimulation with phorbol-12-myristate-13-acetate and ionomycin in the presence of brefeldin and monensin. Similar results were obtained from four individuals at the first trimester of normal pregnancy. **(M–O)** Representative flow cytometric plots **(M)**, bar graphs **(N)** and pie charts **(O)** displaying the proportions of native (T_N_, CD45RO^−^CCR7^+^), effector (T_E_, CD45RO^−^CCR7^−^), T_CM_ (CD45RO^+^CCR7^+^), and T_EM_ (CD45RO^+^CCR7^−^) cells in paired pCD4 and dCD4 T cells. Each symbol reflects a sample and each line reflects the samples from the same person (*n* = 4 per group). pCD4 T, peripheral blood CD4^+^ T; dCD4 T, decidual CD4^+^ T; GO, Gene Ontology; GSEA, gene set enrichment analysis; Nom, Nominal; NES, Normalized Enrichment Score; UD, undetected.

To further characterize the phenotype and functionality of human dCD4 T cells, we performed GSEA analysis for the GO categories involved in cell cycle, activation, proliferation, and cytokine production. Interestingly, we found that the gene sets related to the following categories: M phase of mitotic cell cycle, mitotic sister chromatin segregation, M phase (Figure 4A; Figure S6A in Supplementary Material); lymphocyte activation, T cell activation, regulation of lymphocyte activation (Figure 4B; Figure S6B in Supplementary Material); cell proliferation, (positive) regulation of cell proliferation (Figure 4C; Figure S6C in Supplementary Material); and cytokine production, cytokine secretion and cytokine biosynthetic process (Figure 4D; Figure S6D in Supplementary Material), were remarkably and positively enriched in dCD4 versus pCD4 T cells, indicating that human dCD4 T cells stay in M phase, and show increased activation and proliferation, as well as have an enhanced functionality represented by cytokines production.

Furthermore, we evaluated the transcript and protein abundances of the representative cytokines and “master” transcription factors for the Th1, Th2, Th17, and Treg cell commitment, in resting or activated dCD4 and pCD4 T cells. Data showed that dCD4 T cells had a higher mRNA expression level of *IFNG, IL17A, RORC*, and *FOXP3* at rest, as well as produced more IFN-γ, IL-17A, and Foxp3 upon stimulation with PMA and ionomycin as determined by intracellular staining; in contrast, the mRNA expression of *IL4, IL5, IL13*, and *GATA3* at rest, together with IL-4 secretion after stimulation, were at an extremely low level in both pCD4 and dCD4 T cells or not different between these cells (Figures 4E–L; Figures S7A,B in Supplementary Material). These data suggested that human dCD4 T cells are a heterogeneous population containing Th1, Th17, and Treg cell subsets.

Memory is the hallmark of adaptive immune response, and memory T cells are divided into at least two distinct subsets: central memory T (T_CM_) and effector memory T (T_EM_) cells, based on their different effector functions and homing capacities (42, 51, 52). T_EM_ cells are the first responders capable of migrating into inflamed tissues and possess immediate effector functions, whereas T_CM_ cells can home to lymphoid organs where they readily proliferate and produce more secondary effectors (42, 51). Consistent with previous studies (53, 54), we observed that human dCD4 T cells contained a higher percentage of CD45RO^+^ cells, which are regarded as memory T cells, as compared with pCD4 T cells (Figure S7C in Supplementary Material). Moreover, when we compared the proportions of native (T_N_, CD45RO^−^CCR7^+^), effector (T_E_, CD45RO^−^CCR7^−^), T_CM_ (CD45RO^+^CCR7^+^), and T_EM_ (CD45RO^+^CCR7^−^) cells between pCD4 and dCD4 T cells using flow cytometry staining (51), we found that dCD4 cells significantly increased the proportion of T_EM_ cells but decreased T_N_ cells, revealing that human dCD4 T cells mainly consist of T_EM_ cells whereas T_N_ cells are almost absent (Figures 4M–O).

Collectively, these results showed that human dCD4 T cells during early pregnancy are endowed with enhanced activation, high proliferation potential, and elevated functionality in terms of cytokine production, as well as with a complex nature containing Th1, Th17, and Treg cell subsets and displaying an effector-memory phenotype.

### Genes in dCD4 T Cells Undergo a Comparable Number of Upregulated and Downregulated AS Events

**Figure 5:**
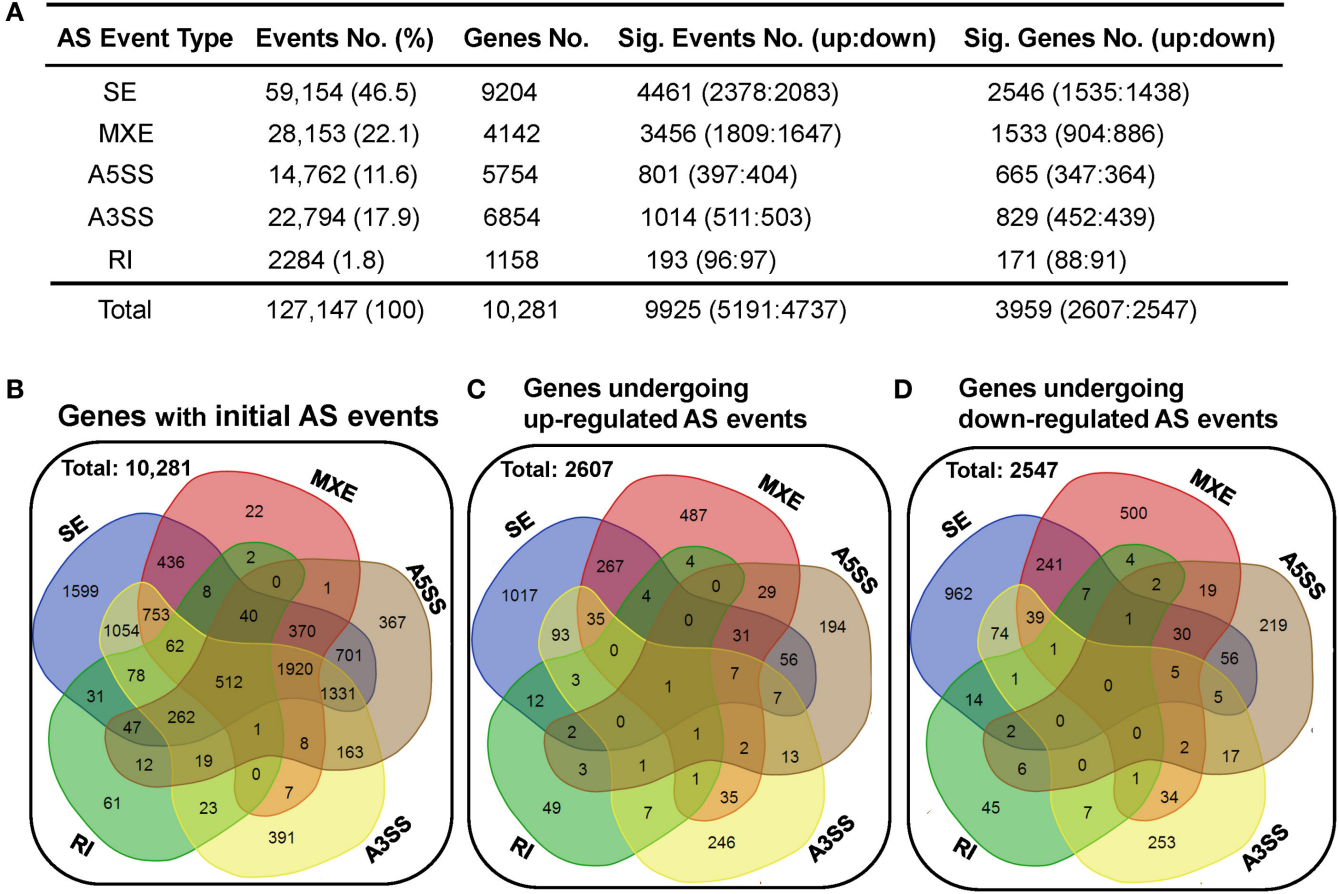
dCD4 T cells undergo a comparable number of upregulated and downregulated AS events. **(A)** Summary of the numbers of five types of AS events, differentially expressed events (with upregulated and downregulated events) and the genes they belong to. Column 1 shows the five most common types of AS events that were investigated. Columns 2 and 3 show the initial number of AS events found in the combined samples of pCD4 and dCD4 T cells with their frequency among total events (column 2), as well as the number of genes these events belong to (column 3). Columns 4 and 5 show the number of differentially expressed events (FDR < 0.05 with |ΔΨ| > 0.05 between samples) with upregulated (FDR < 0.05 with ΔΨ > 0.05) and downregulated (FDR < 0.05 with ΔΨ < −0.05) AS events in dCD4 T cells versus their autologous pCD4 T counterparts (column 4), as well as the number of genes that these significant events belong to (column 5). **(B)** Venn diagram of genes showing the initial SE, MXE, A5SS, A3SS, and RI in the combined samples (pCD4 and dCD4 T cells). **(C,D)** Venn diagram of genes showing upregulated **(C)** or down-regulated **(D)** SE, MXE, A5SS, A3SS, and RI events in dCD4 T cells with respect to pCD4 T cells. AS, alternative splicing; SE, skipped exon; MXE, mutually exclusion exons; A5SS, alternative 5′ splice site; A3SS, alternative 3′ splice site; RI, retained intron; %, percentage; No., Number; Sig., Significant; up, upregulated; down, downregulated; FDR, false discovery rate.

Alternative splicing is an important mechanism involved in shaping CD4 T-cell activation, differentiation, and immune response to stimulation (30–33). Here, we applied the rMATS (v3.2.1 beta) paired model to identify and analyze the differentially expressed AS events using the splice junction counts as the input (46). Five basic and generally recognized AS modes were investigated, including skipped exon (SE), mutually exclusion exons (MXE), alternative 5′ splice site (A5SS), alternative 3′ splice site (A3SS), and retained intron (RI). A total of 127,147 AS events, belonging to 10,281 genes, were found in the dCD4 and pCD4 T cells, with 512 genes showing evidence of all five AS types (Figures 5A,B). SE was the most common type of AS event, accounting for 46.5% (59,154 SE events in 9,204 genes) of all splicing events; in contrast, RI was the least common (1.8%, 2,284 RI events in 1,158 genes) (Figures 5A,B).

Interestingly, compared with pCD4 T cells, comparable numbers of upregulated (at the threshold of FDR < 0.05 and ΔΨ > 0.05) and downregulated (FDR < 0.05 and ΔΨ < −0.05) splicing events were observed in human dCD4 T cells (upregulated events versus downregulated events): 2,378 versus 2,083 of SE, 1,809 versus 1,647 of MXE, 397 versus 404 of A5SS, 511 versus 503 of A3SS, 96 versus 97 of RI, and 5,191 versus 4,737 of total AS events (Figure 5A; Tables S2–S6 in Supplementary Material). These differential AS events also occurred in a comparable number of genes (genes showing upregulated events versus downregulated events): 1,535 versus 1,438 for SE, 904 versus 886 for MXE, 347 versus 364 for A5SS, 452 versus 439 for A3SS, 88 versus 91 for RI, and 2,607 versus 2,547 for all AS events (Figures 5A,C,D). In addition, the numbers of genes undergoing SE-, MXE-, A5SS-, A3SS-, or RI-upregulation specifically, or of genes undergoing two or more types’ upregulation simultaneously, were comparable to those undergoing downregulation specifically or simultaneously, in dCD4 versus pCD4 T cells (Figures 5C,D).

### Both Upregulated and Downregulated AS Events in dCD4 T Cells Are Enriched in the Genes Related to Cellular Metabolic Process

**Figure 6:**
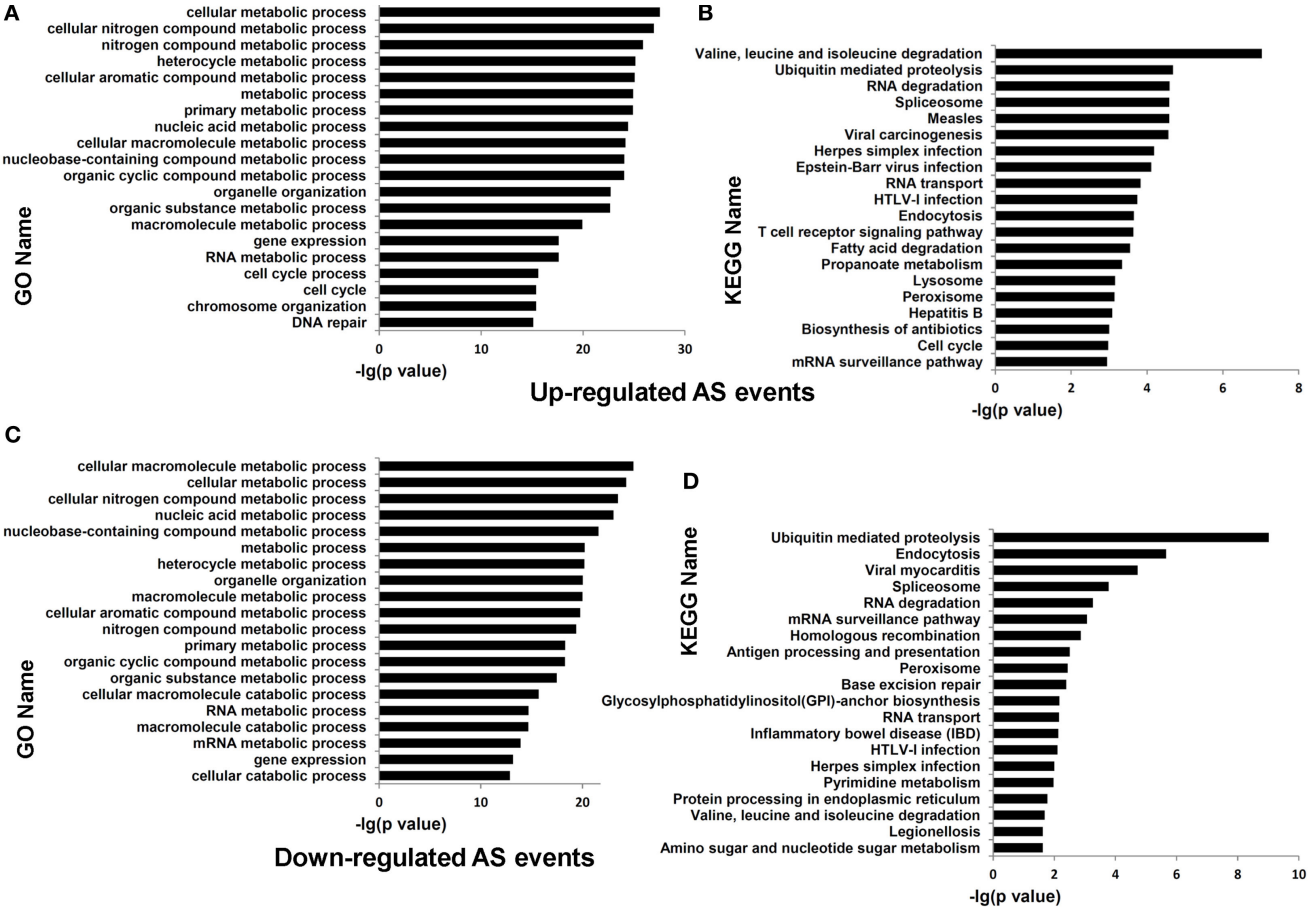
Functional enrichment analysis of the genes undergoing upregulated or downregulated AS events in dCD4 T cells. **(A–D)** The top 20 GO **(A,C)** and KEGG **(B,D)** terms enriched for genes undergoing upregulated [**(A,B)**, FDR < 0.05 with ΔΨ > 0.05 between samples] or downregulated [**(C,D)**, FDR < 0.05 with ΔΨ < −0.05 between samples] AS events (SE, MXE, A5SS, A3SS, and RI are combined together) in dCD4 versus pCD4 T cells. AS, alternative splicing; GO, Gene Ontology; KEGG, Kyoto Encyclopedia of Genes and Genomes; FDR, false discovery rate; SE, skipped exon; MXE, mutually exclusion exons; A5SS, alternative 5′ splice site; A3SS, alternative 3′ splice site; RI, retained intron.

Functional enrichment analysis of the genes undergoing differential AS events in dCD4 T cells (at the threshold of |ΔΨ| > 0.05 and FDR < 0.05, compared with pCD4 T cells; including all five AS types) highlighted the categories related to cellular metabolic process (*P* = 4.12E−43 with GO annotation), cellular nitrogen compound metabolic process (*P* = 3.85E−40), cellular macromolecule metabolic process (*P* = 1.18E−38), ubiquitin mediated proteolysis (*P* = 4.00E−10 with KEGG analysis), valine, leucine, and isoleucine degradation (*P* = 7.69E−09), and the spliceosome (*P* = 5.95E−07) (Figure S8 in Supplementary Material). Furthermore, enrichment analysis of genes containing upregulated AS events (at the threshold of ΔΨ > 0.05 and FDR < 0.05, compared with pCD4 T cells; including all five AS types) highlighted the categories related to cellular metabolic process (*P* = 2.78E−28 with GO annotation), cellular nitrogen compound metabolic process (*P* = 1.08E−27), cellular macromolecule metabolic process (*P* = 6.77E−25), ubiquitin mediated proteolysis (*P* = 2.07E−05 with KEGG analysis), RNA degradation (*P* = 2.54E−05), and the spliceosome (P = 2.60E−06), which were also significantly enriched for the genes containing downregulated AS events (at the threshold of ΔΨ < −0.05 and FDR < 0.05; *P* = 4.94E−25, 3.21E−24, 9.60E−26, 9.64E−10, 5.47E−04, and 1.68E−04, respectively) in human dCD4 T cells (Figure 6). Additionally, enrichment analysis of the genes undergoing only upregulated or only downregulated AS events, or the genes showing evidence of both upregulated and downregulated AS events simultaneously, also highlighted the term related to cellular metabolic process (Figures S9–S10 in Supplementary Material). Similar results were observed when dividing AS events into SE, MXE, A5SS, A3SS, and RI, respectively (Figures S11–S20 in Supplementary Material). Therefore, these data revealed that both upregulated and downregulated AS events in dCD4 T cells are remarkably enriched in the genes related to cellular metabolic process.

### Changes at the AS Event Level Do Not Imply Measurable Differences at the Gene Expression Level in dCD4 T Cells

**Figure 7:**
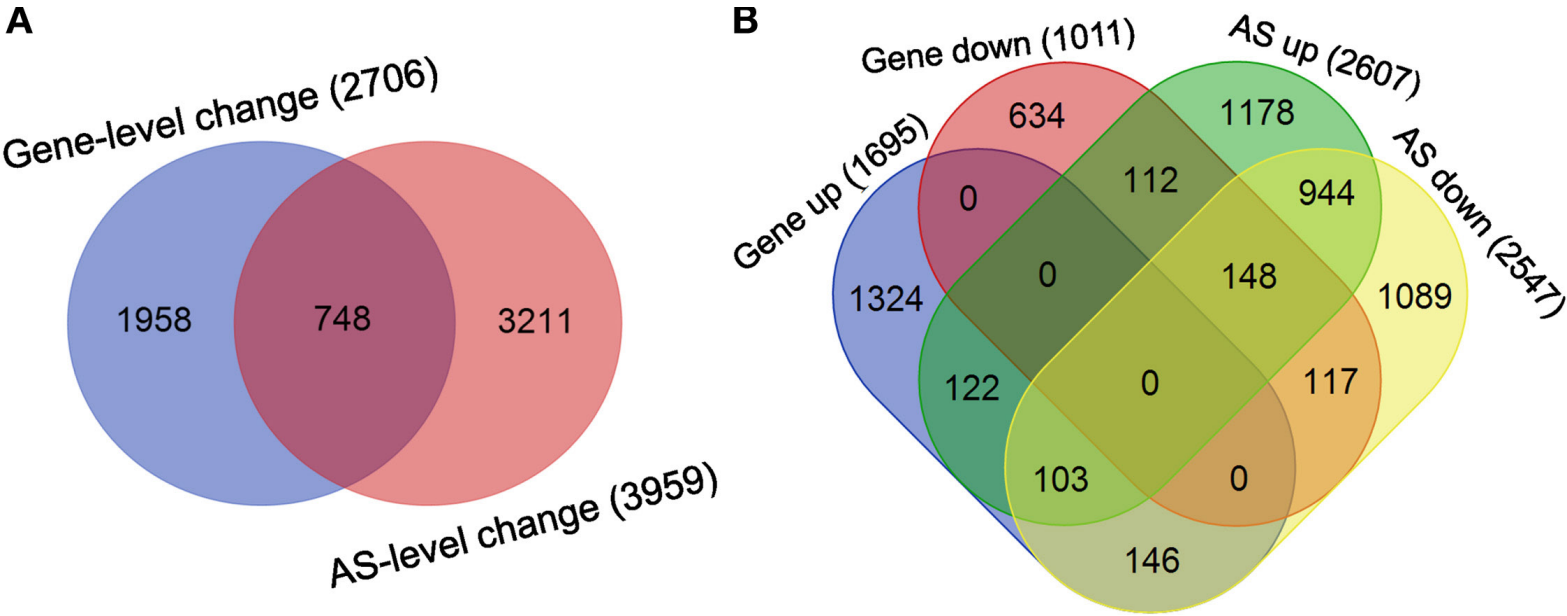
Changes at the AS event level do not imply measurable differences in the gene expression level in dCD4 T cells. **(A)** Venn diagram of genes undergoing significant splicing-level and expression-level changes in dCD4 T cells with respect to pCD4 T cells. **(B)** Venn diagram of genes undergoing splicing-level upregulation and down-regulation, as well as expression-level upregulation and downregulation, in dCD4 T cells with respect to pCD4 T cells. AS, alternative splicing; up, upregulated; down, downregulated.

Finally, the overlap between the sets of genes undergoing differential splicing-level and expression-level changes in dCD4 T cells was observed to be very low: 748 genes in the overlap versus 2,706 genes showing expression-level changes and 3,959 genes showing splicing-level changes (Figure 7A). Meanwhile, the overlap between sets of genes undergoing splicing-level and expression-level upregulation or downregulation in dCD4 T cells was also very low: 122 genes in the overlap versus 1,695 genes showing expression-level upregulation and 2,607 genes showing splicing-level upregulation; 117 genes in the overlap versus 1,011 genes showing expression-level downregulation and 2,547 genes showing splicing-level downregulation (Figure 7B). These results indicated that changes at the AS event level do not result in measurable differences at the gene expression level in human dCD4 T cells.

## Discussion

T cells possess a particular capacity to capture membrane molecules from APCs through a process termed trogocytosis (55–58). Zhou et al. revealed that presentation of captured peptide-MHC-II ligands by CD4^+^ T cells has a stimulatory effect on naive T cells, and these cells with acquired peptide-MHC-II molecules become effector CD4^+^ T cells that manifest a better recall response (56). In the present study, human dCD4 T cells in early pregnancy were found to significantly upregulate the genes involved in immune response (Figure 1) and antigen processing and presentation (Figure S4 in Supplementary Material), with an upregulation of most MHC-II molecules including *HLA-DMA, DMB, DOA, DPA1, DPB1, DQA1, DQA2, DQB1, DQB2, DRA, DRB1, DRB5*, and *DRB6* (Figure S21 in Supplementary Material), suggesting that human dCD4 T cells have acquired MHC-II molecules and become effector cells that provide a better immune response than pCD4 T cells during early pregnancy.

Since the first trimester of pregnancy is believed to be a pro-inflammatory phase, the presence of immune cells in the decidua, which are active, functional and carefully controlled rather than being suppressed, is important for a healthy pregnancy outcome (59, 60). Indeed, human dCD4 T cells were reported to express multiple T-cell-activation surface markers during early pregnancy, such as the early activation antigen CD69, late activation antigen HLA-DR and very late antigen 1 (also known as ITGA1 and CD49a), indicating that these cells are regionally activated (17, 61–66). Consistent with that, our data from genome-wide transcriptional profiling showed that the genes related to T-cell activation are highly enriched, with most of them being upregulated in dCD4 T cells (Figure 4B; Figure S6B in Supplementary Material), such as *CD25, CD38, CD69, CD122, ITGA1* (*CD49a*), *LAT2, CD40LG*, and *HLA-DRs* (Figure 2; Figure S21 in Supplementary Material), suggesting that human dCD4 T cells are more active than their counterparts in the peripheral blood. In addition, dCD4 T cells expressed a high level of proliferation-associated gene *MKI67* (encoding antigen Ki-67) (Figure S21G in Supplementary Material), and were found to be at M phase and show increased proliferation and functionality in terms of cytokine production (Figure 4). However, the mechanisms beyond this enhanced profile of human dCD4 T cells during early pregnancy remain poorly understood and require further investigation.

Herein, human dCD4 T cells were observed to highly upregulate both transcript and protein levels of chemokine receptors CXCR3 and CCR6 (Figure 2), which are preferentially expressed on Th1 and Th17 cells, respectively (67, 68). CXCR3^+^CCR6^+^CD4^+^ cells were reported to be enriched in conventional Th1 and Th17 cells, as well as Th1/17 cells that are characterized by their capacity to co-produce IFN-γ and IL-17, with conventional Th1 cells being dominantly enriched (69). Our co-expression analysis revealed that both CXCR3^+^CCR6^+^ and CXCR3^+^CCR6^−^ cells are increased in dCD4 versus pCD4 T cells (Figure S22 in Supplementary Material), indicating that dCD4 T cells probably contain more Th1, Th17, and/or Th1/17 cells. Indeed, we found that human dCD4 T cells can produce more IFN-γ, IL-17A, and even Foxp3, but not IL-4, upon stimulation with PMA and ionomycin (Figures 4E–M). However, the cells co-producing IFN-γ/IL-17A (IFN-γ^+^IL-17A^+^, Th1/17 cells), IFN-γ/IL-4 (IFN-γ^+^IL-4^+^), IFN-γ/Foxp3 (IFN-γ^+^Foxp3^+^), IL-17A/IL-4 (IL-17A^+^IL-4^+^), IL-17A/Foxp3 (IL-17A^+^Foxp3^+^), or IL-4/Foxp3 (IL-4^+^Foxp3^+^), were very few or undetectable in both pCD4 and dCD4 T cells (Figure S22 in Supplementary Material). Therefore, our data showed that human dCD4 T cells are a heterogeneous population containing conventional Th1, Th17, and Treg cell subsets, whereas the cells co-producing IFN-γ, IL-17A, or Foxp3 are almost absent. These findings are suggestive of a potential role of these subsets in a successful pregnancy.

At present, a large body of evidence has demonstrated that decidual Treg cells contribute to creating a local tolerant microenvironment and maintaining a successful pregnancy; however, the role and importance of other Th subsets in the decidua is still largely unknown or remains controversial (3, 16, 23, 24, 70). Consistent with our results (Figures 4E–L), it was reported that IFN-γ and IL-17, secreted by human decidual Th1 and Th17 cells, play an important role in vascular remodeling (71) and trophoblast invasion (23), and that Th2 cytokines are not prerequisite to maintain a normal pregnancy. Nonetheless, a study using chemokine receptor expression profiles showed that while Tregs cells are significantly enriched in the decidua of early pregnant women, Th17 cells are nearly absent (13). Moreover, our results are in contrast with the hypothesis that there is a suppressed Th1- and Th17-type immunity associated with a predominant Th2-type immunity during normal pregnancy (60, 71–73). Whether the presence of Th1 and Th17 cells in decidua is required or not for a healthy pregnancy outcome needs further investigation.

T-bet is the “master” transcription regulator that directs Th1-cell lineage commitment through transactivation of IFN-γ (74). However, previous studies showed that rapid IFN-γ production by memory CD4^+^ T cells occurred in the absence of T-bet expression (75); the level of T-bet peaked only and briefly when Ifng^+^ CD4 T_E_ expanded (76). Moreover, in the memory phase, only a small percentage of Ifng^+^ T cells maintained a T-bet^hi^ phenotype (76); and downregulation of T-bet was required for the development of tissue-resident memory T cells in epithelial sites (77). Consistent with these findings, we observed that when human dCD4 T cells produced a higher level of IFN-γ and displayed a memory phenotype (Figure 4), there was no difference in *TBX21* (encoding T-bet) expression between pCD4 and dCD4 T cells (Figure S23 in Supplementary Material).

In eukaryotic organisms, most pre-mRNAs tend to undergo AS, which is a ubiquitous and vital mechanism to control gene expression at the co- and post-transcriptional level, resulting in production of multiple distinct mRNA transcripts and proteins with diverse cellular functions (25, 78). Blencowe and colleagues performed the first analysis of AS complexity in human tissues using high-throughput mRNA-Seq datasets (consisting of 17–32 million 32-bp-long reads), estimating that there are approximately 100,000 AS events in major human tissues, with an intermediate- to high-abundance (35). After aligning all the sequenced reads (approximately 30–40 million 2 × 125-bp paired-end-long reads) to the human reference genome (hg19 version), between 88 and 93% of reads per sample were uniquely mapped (Figure 1A), and a total of 127,147 AS events, belonging to 10,281 genes, were also found in human dCD4 and pCD4 T cells during early pregnancy (Figure 5A). Furthermore, our data revealed that SE is the most common type of AS event (46.5%), whereas RI is the least common (1.8%) in human dCD4 and pCD4 T cells (Figure 5A). These results are consistent with the observations in human hematopoietic progenitor cells (26), human embryonic kidney 293 T and Ramos B cells (79), and human epithelial (PC3E) and mesenchymal (GS689) prostate cancer cell lines (46); however, these results differ from the findings in *Physcomitrella* (80), *Arabidopsis* (81), rice (82), filamentous fungus *Trichoderma longibrachiatum* (83) and *Verticillium dahliae* (84), where RI is the most prevalent mechanism, and also differ from the observation in bovine longissimus muscle (36) where A3SS is the primary AS mode.

Using quantitative AS microarray profiling, recent studies have shown that the majority of the genes undergoing differential AS-level changes are different from the set of genes showing differential expression-level changes during Jurkat T-cell activation (33), and many more genes are affected through AS modulation than being affected by expression-level modulation in CD4^+^ T cells upon CD28 co-stimulation (85). Similarly, the overlap between the sets of genes undergoing differential splicing-level and expression-level changes in dCD4 T cells was observed to be very low in our study (Figure 7A). Functional enrichment analysis revealed that the set of genes with changes in both expression and AS levels (748 genes in the overlap) is enriched in immune system process, which is distinct from the observation in the genes undergoing only AS-level change (3,211 gene, enriched in cellular metabolic process) but similar with those genes showing only expression-level change (1,958 genes, enriched in immune system process) (Figure S24 in Supplementary Material). Since the upregulated genes in dCD4 T cells were also significantly enriched in immune system process (Figure 1), we speculated that AS might play a key role in regulating the expression of these upregulated genes. However, our data showed that the overlap between sets of genes undergoing splicing-level and expression-level upregulation was very low: 122 genes in the overlap versus 1,695 genes showing expression-level upregulation and 2,607 genes showing splicing-level upregulation (Figure 7B). Moreover, although dCD4 T cells expressed a higher level of *IFNG, CXCR3, RORC, IL17A, CCR6, FOXP3, IL-10, PDCD1*, and *CCR7* (Figures 2 and 4; Figures S5, S7, and S23 in Supplementary Material), which are important genes for CD4^+^ T-cell differentiation, function or recruitment, the total and differentially expressed AS events in these genes were surprisingly few (*IFNG, CXCR3, RORC, PDCD1, CCR7*) or undetectable (*IL17A, IL-10*) in both pCD4 and dCD4 T cells, or not different between these cell populations (*CCR6, FOXP3*) (Table S7 in Supplementary Material). These data indicated that AS does not make a major contribution to regulate gene expression of these key molecules, and that changes at the AS event level do not imply measurable differences at the gene expression level in human dCD4 T cells.

In summary, we have examined the transcriptional and AS signatures of paired dCD4 pCD4 T cells from healthy women at the first trimester of normal pregnancy. Our data revealed that dCD4 and pCD4 T cells show a distinct transcriptional signature: dCD4 T cells upregulate genes involved in the immune system process, but downregulate genes related to mRNA catabolic process and the ribosome; human dCD4 T cells stay in M phase, and show increased activation, proliferation, and cytokine production, as well as contain Th1, Th17, and Treg cell subsets and display an effector-memory phenotype. However, dCD4 T cells undergo a comparable number of upregulated and downregulated AS events, both of which are enriched in the genes related to cellular metabolic process. Moreover, the changes at the AS event level do not reflect measurable differences at the gene expression level in dCD4 T cells. This study thus provides a comprehensive framework of the transcriptional and AS landscapes of human dCD4 T cells, which deepens our understanding of the characteristic and functionality of these cells during early pregnancy.

## Ethics Statement

This study was approved and performed in compliance with the Medical Ethics Committee of the International Peace Maternity and Child Health Hospital of China Welfare Institute (Shanghai, China). Informed consent was obtained from all participants in written form before enrollment.

## Author Contributions

WZ and ZL designed and performed most of the experiments and drafted the manuscript. XL, SZ, YZ, XM, TY, FT, X-RL, and JF performed a part of the experiments. AK and WZ edited the manuscript. YL supervised the study and proofread the manuscript.

## Conflict of Interest Statement

The authors declare that the research was conducted in the absence of any commercial or financial relationships that could be construed as a potential conflict of interest.

## Abbreviations

AS, alternative splicing; mRNA-Seq, mRNA sequencing; dCD4 T, decidual CD4^+^ T; pCD4 T, peripheral blood CD4^+^ T; Treg, T regulatory; MFI, maternal-fetal interface; PBMCs, peripheral blood mononuclear cells; GO, Gene Ontology; KEGG, Kyoto Encyclopedia of Genes and Genomes; GSEA, gene set enrichment analysis; FDR, false discovery rate; NES, Normalized Enrichment Score; APCs, antigen-presenting cells; SE, skipped exon; MXE, mutually exclusion exons; A5SS, alternative 5′ splice site; A3SS, alternative 3′ splice site; RI, retained intron.
