# Supplementary material for: Distinct Transcriptional and Alternative Splicing Signatures of Decidual CD4+ T Cells in Early Human Pregnancy
Source: Front Immunol. 2017 Jun 12;8:682. doi: 10.3389/fimmu.2017.00682 (PMC5466981; doi:10.3389/fimmu.2017.00682)
Supplement: Supplementary file 8 [file Image_1.PDF]

Supplementary Materials

Title: Distinct Transcriptional and Alternative Splicing Signatures for Decidual CD4$^+$ T Cells in Early Human Pregnancy

Authors:

Weihong Zeng[1,#], Zhicui Liu[2,#], Xinmei Liu[1], Siming Zhang[1], Asma Khanniche[3], Ying Zheng[4], Asma Khanncihe[4], Xiaoling Ma[1], Tiantian Yu[1], Fuju Tian[1], Xiao-Rui Liu[1], Jianxia Fan[1] and Yi Lin[1,*]

Institution:

[1] Institute of Embryo-Fetal Original Adult Disease Affiliated to Shanghai Jiao Tong University School of Medicine, the International Peace Maternity & Child Health Hospital, Shanghai Jiao Tong University School of Medicine, Shanghai 200030, P. R. China.

[2] Department of Dermatology, Ruijin Hospital, Shanghai Jiaotong University School of Medicine, Shanghai 200025, P. R. China.

[3] Shanghai Institute of Imunology, Shanghai Jiao Tong University School of Medicine, Shanghai 200025, P. R. China.

[4] Out-patient Operatingroom, the International Peace Maternity & Child Health Hospital, Shanghai Jiao Tong University School of Medicine, Shanghai 200030, P. R. China.

[#] These authors contributed equally to the study.

[*] **Corresponding author:**

Yi Lin, Institute of Embryo-Fetal Original Adult Disease Affiliated to Shanghai Jiao Tong University School of Medicine, the International Peace Maternity & Child Health Hospital, Shanghai Jiao Tong University School of Medicine, No. 910, Hengshan Road, Shanghai 200030, P. R. China. Telephone: +86-21-64070434. Fax: +86-21-64073421. E-mail: yilinonline@126.com.

Running title: Transcriptome of decidual CD4$^+$ T cells

Supplementary Figures

A

Group 1: Samples for mRNA-Seq

Sample	Age (years)	Gestational time (Days)
1	28	47
2	22	44
3	27	58

B

Group 2: Samples for validation of RNA-Seq data by flow cytometry staining

Sample	Age (years)	Gestational time (Days)
1	39	42
2	28	50
3	28	50
4	33	38
5	22	42

C

Group 3: Samples for $CD4^+$ T-cell functional status analysis

Sample	Age (years)	Gestational time (Days)
1	35	43
2	34	50
3	39	43
4	30	43

Figure S1. Summary of the information regarding samples used for mRNA sequencing (mRNA-Seq, A), validation of RNA-Seq data by flow cytometry staining (B) and $CD4^+$ T-cell functional status analysis (C).

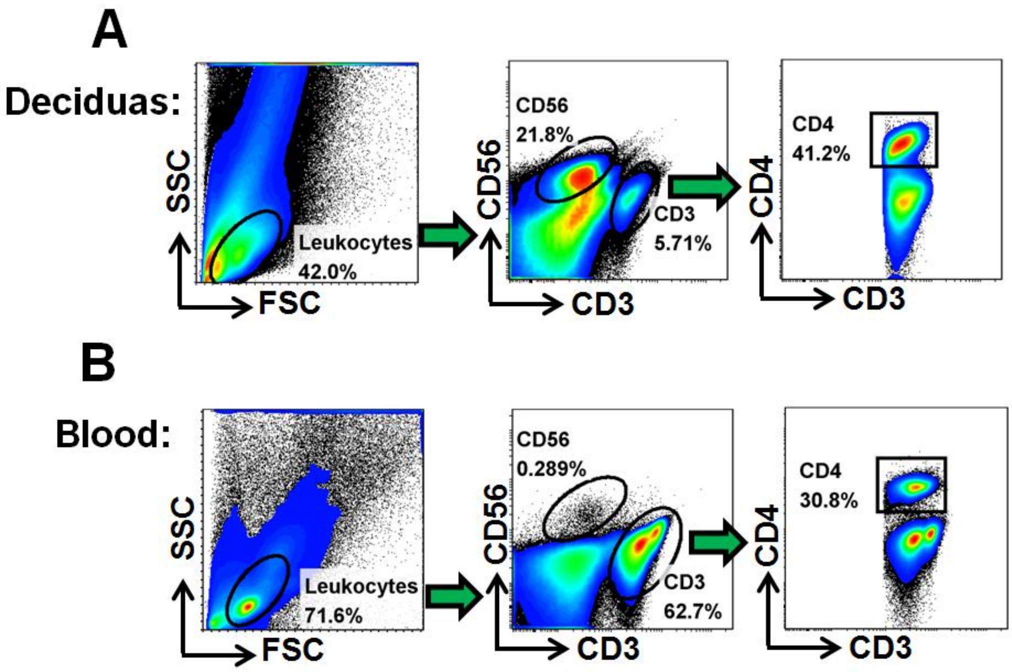

Figure S2. Flow cytometric plots illustrate the gating strategy used to identify and isolate the $CD4^+$ T cells in paired decidual (A) and peripheral blood (B) samples by fluorescence-activated cell sorting (FACS), based on the phenotype of $CD56^-CD3^+CD4^+$.

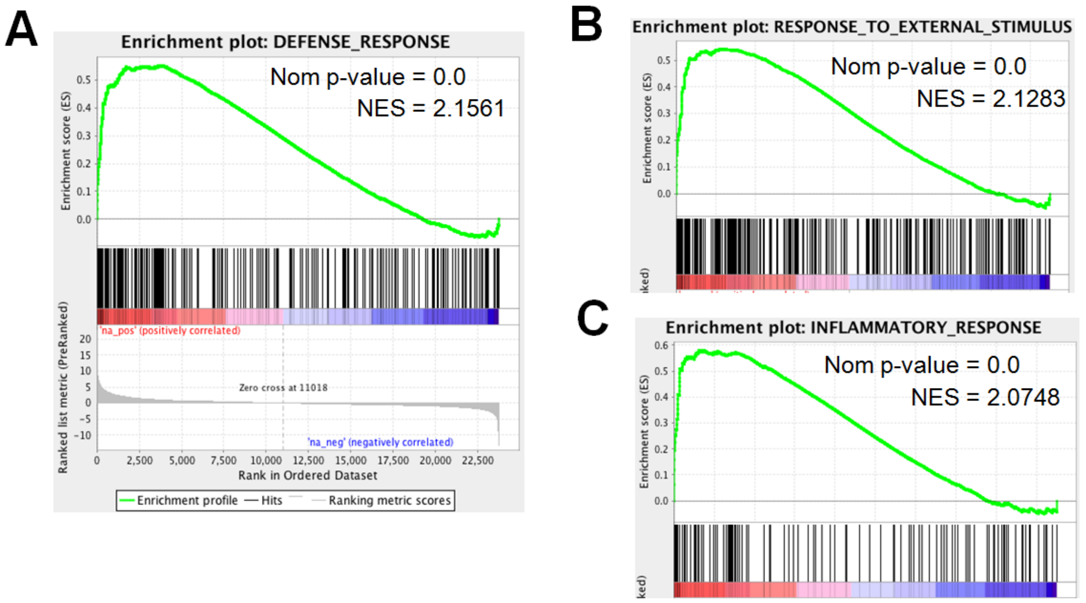

Figure S3. GSEA plots of GO categories including defense response (A), response to external stimulus (B) and inflammatory response (C) in dCD4 *versus* pCD4 T cells. GSEA, Gene Set Enrichment Analysis; GO, Gene Ontology; Nom, Nominal; NES, Normalized Enrichment Score.

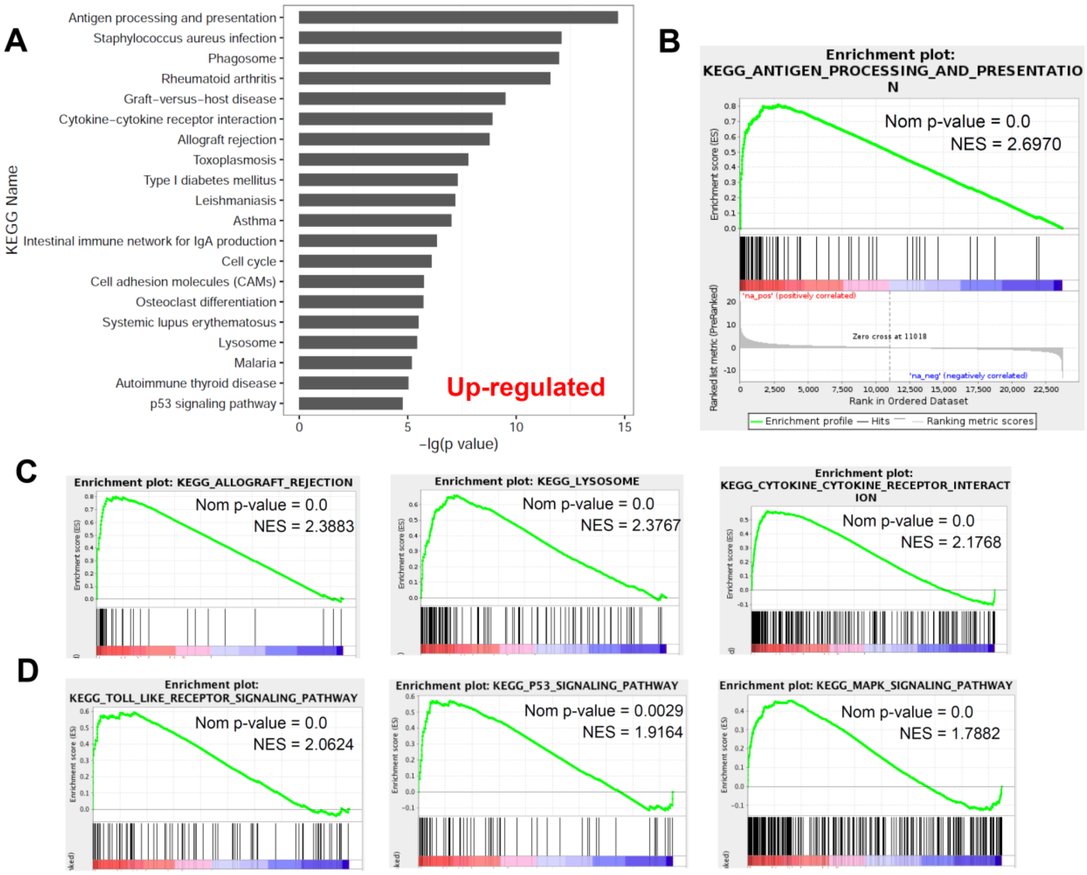

Figure S4. KEGG pathway analysis of genes upregulated in dCD4 T cells and GSEA plot of KEGG categories. (A) The top 20 KEGG terms enriched for the upregulated genes in dCD4 T cells. **(B-D)** GSEA plots of KEGG categories including antigen processing and presentation (**B**), allograft rejection, lysosome, cytokine cytokine receptor interaction (**C**), Toll like receptor signaling pathway, and p53 and MAPK signaling pathways (**D**), in dCD4 T *versus* pCD4 T cells. KEGG: Kyoto Encyclopedia of Genes and Genomes; GSEA: Gene Set Enrichment Analysis; Nom, Nominal; NES, Normalized Enrichment Score.

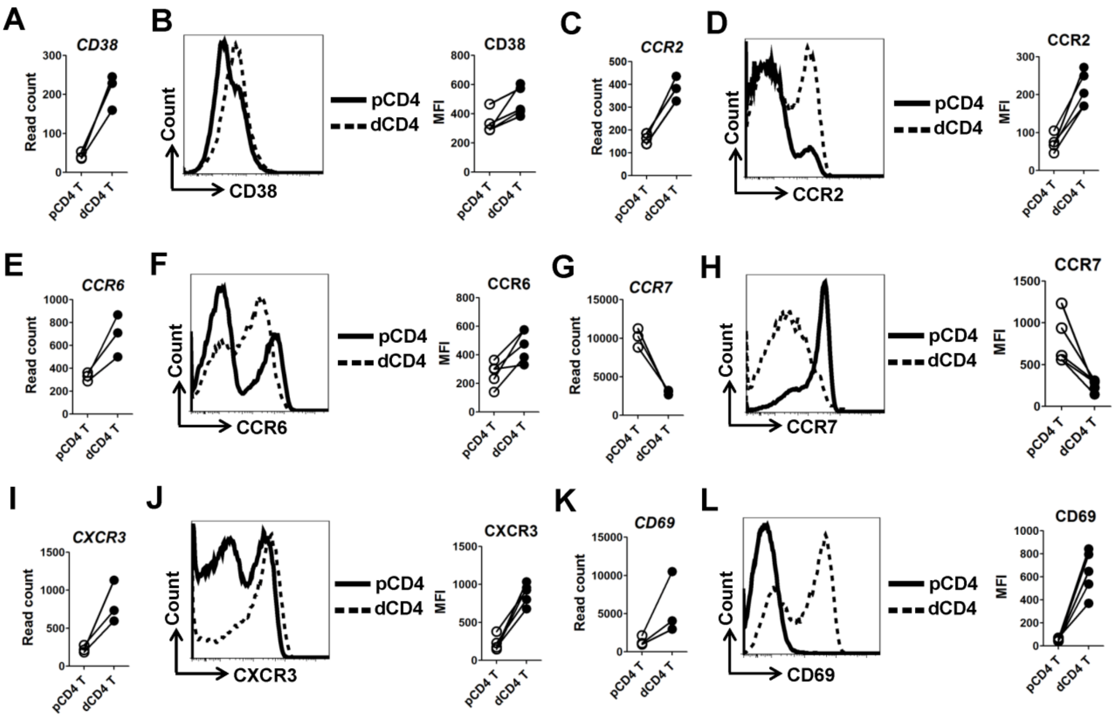

Figure S5. Confirmation of mRNA-Seq data by flow cytometry staining. (A, C, E, G, I and K) Comparison of the gene expression (measured as the read count) of *CD38*, *CCR2*, *CCR6*, *CCR7*, *CXCR3* and *CD69* between in paired pCD4 and dCD4 T cells. Each symbol reflects a sample and each line reflects the samples from the same individual (n = 3 per group). **(B, D, F, H, J and L)** Representative flow cytometry histograms (left) and cumulative data (right) illustrating the comparison of the molecular expression of indicated proteins between paired pCD4 and dCD4 T cells (n = 5 per group). The cells were gated in $CD4^{+}$ T cells and the geometric MFI values were calculated using FlowJo 7.6.1 software. pCD4 T, peripheral blood $CD4^{+}$ T; dCD4 T, decidual $CD4^{+}$ T; %, percentage; MFI, mean fluorescent intensity.

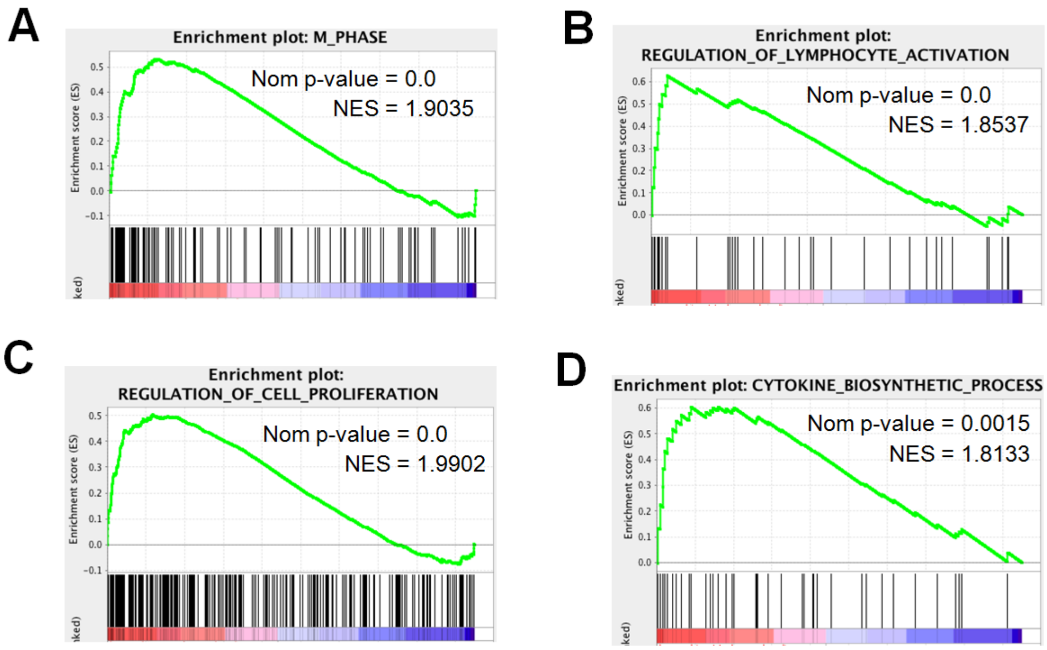

Figure S6. GSEA plots of GO categories including M phase (A), regulation of lymphocyte activation (B), regulation of cell proliferation (C) and cytokine biosynthetic process (D) in dCD4 *versus* pCD4 T cells. GSEA, Gene Set Enrichment Analysis; GO, Gene Ontology; Nom, Nominal; NES, Normalized Enrichment Score.

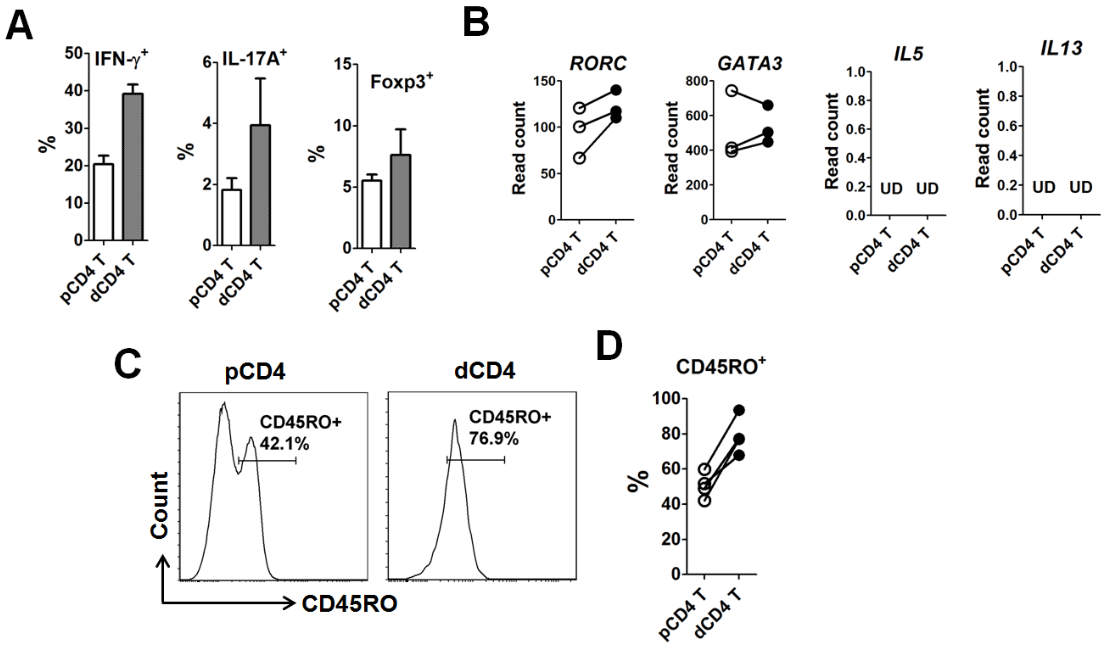

Figure S7. Human dCD4 T cells are a heterogeneous population containing Th1, Th17 and Treg cell subsets, and display a memory phenotype. (A) Comparison of the IFN-γ, IL-17A and Foxp3 expression in $CD4^+$ T cells between decidual (dCD4 T) and peripheral blood (pCD4 T) samples as determined by intracellular staining upon stimulation with PMA and ionomycin (n = 4 per group). **(B)** Comparison of the gene expression (measured as the read count) of *RORC*, *GATA3*, *IL5* and *IL13* between in paired pCD4 and dCD4 T cells in resting (n = 3 per group). **(C-D)** Representative flow cytometric histograms **(C)** and cumulative data **(D)** illustrating the comparison of CD45RO expression between on paired pCD4 and dCD4 T cells (n = 4 per group). The cells were gated in $CD4^+$ T cells. Each symbol reflects a sample and each line reflects the samples from an individual. dCD4 T, decidual $CD4^+$ T; pCD4 T, peripheral blood $CD4^+$ T; %, percentage.

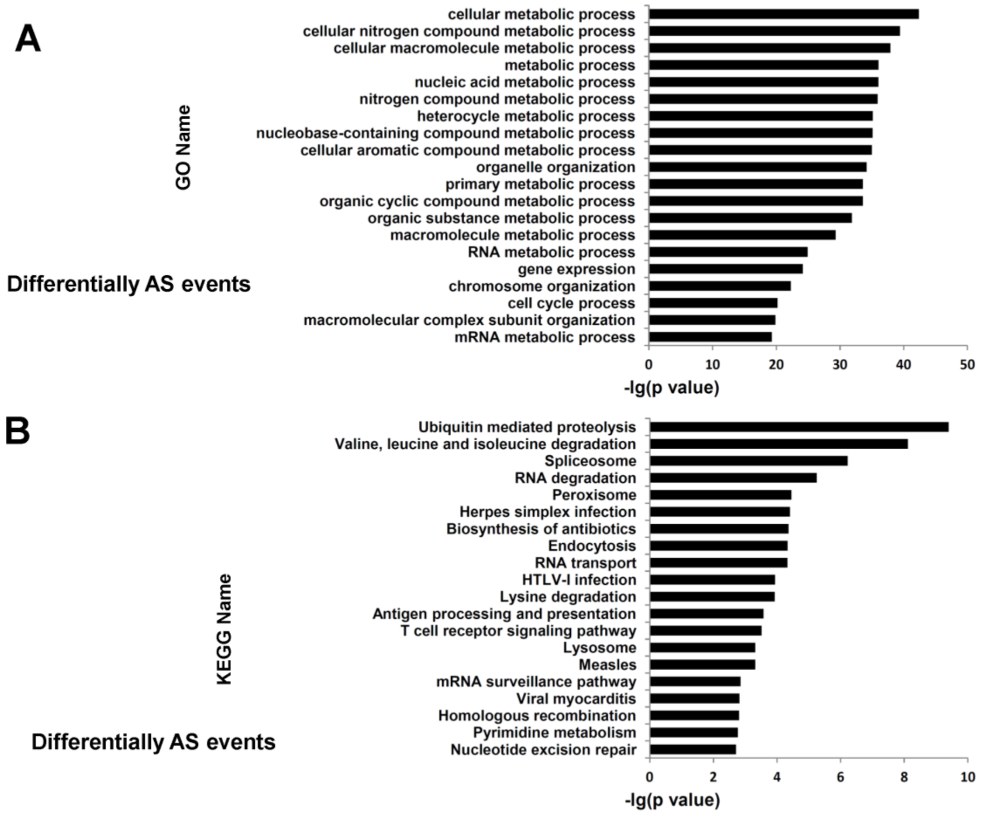

Figure S8. Functional enrichment analysis of the genes undergoing differential alternative splicing (AS) events in dCD4 T cells. (A-B) The top 20 GO (**A**) and KEGG (**B**) terms enriched for genes undergoing differentially expressed AS events (FDR < 0.05 with |ΔΨ| > 0.05 between samples; SE, MXE, A5SS, A3SS and RI are combined together) in dCD4 T *versus* pCD4 T cells. GO, Gene Ontology; KEGG, Kyoto Encyclopedia of Genes and Genomes; SE, skipped exon; MXE, mutually exclusion exons; A5SS, alternative 5' splice site; A3SS, alternative 3' splice site; RI, retained intron.

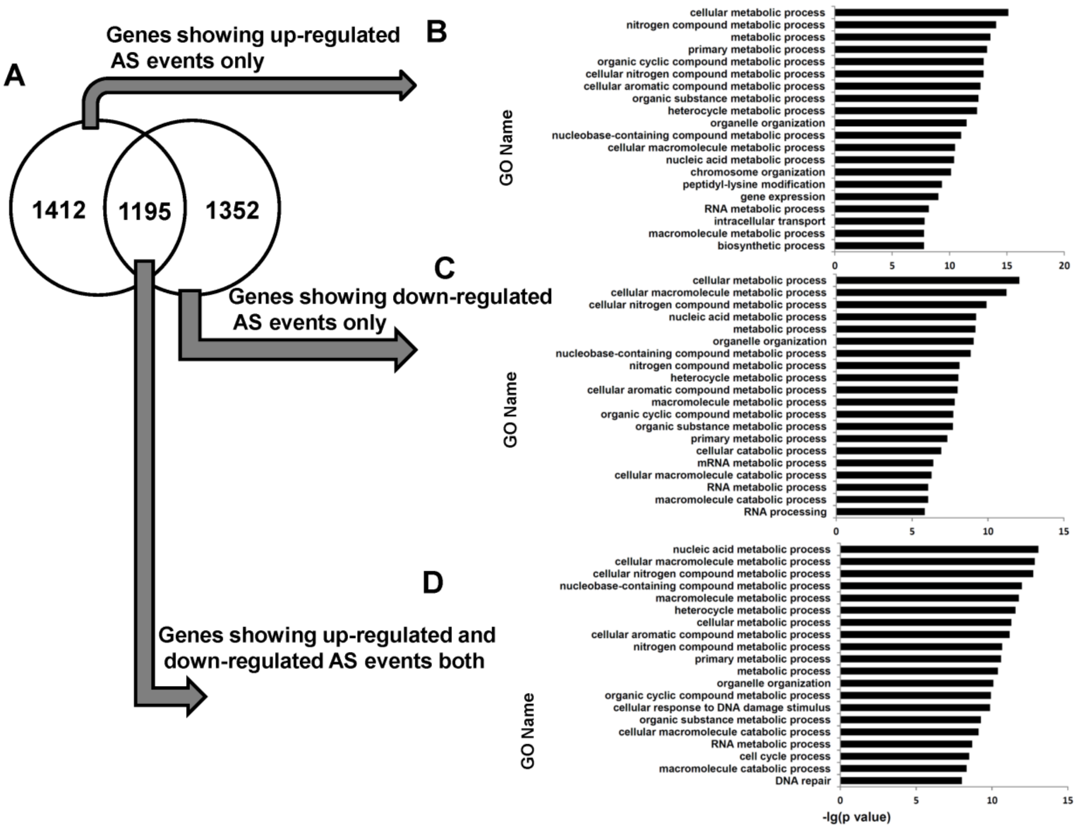

Figure S9. Venn diagram and GO annotation for the genes undergoing upregulated and downregulated AS events in dCD4 T cells. (A-D) Venn diagram (**A**) and GO annotation analysis (**B-D**) for genes undergoing upregulated (FDR < 0.05 with ΔΨ > 0.05 between samples) and downregulated (FDR < 0.05 with ΔΨ < -0.05 between samples) AS events (SE, MXE, A5SS, A3SS and RI are combined together) in dCD4 T cells with respect to pCD4 T cells. GO, Gene Ontology; FDR, false discovery rate; SE, skipped exon; MXE, mutually exclusion exons; A5SS, alternative 5' splice site; A3SS, alternative 3' splice site; RI, retained intron.

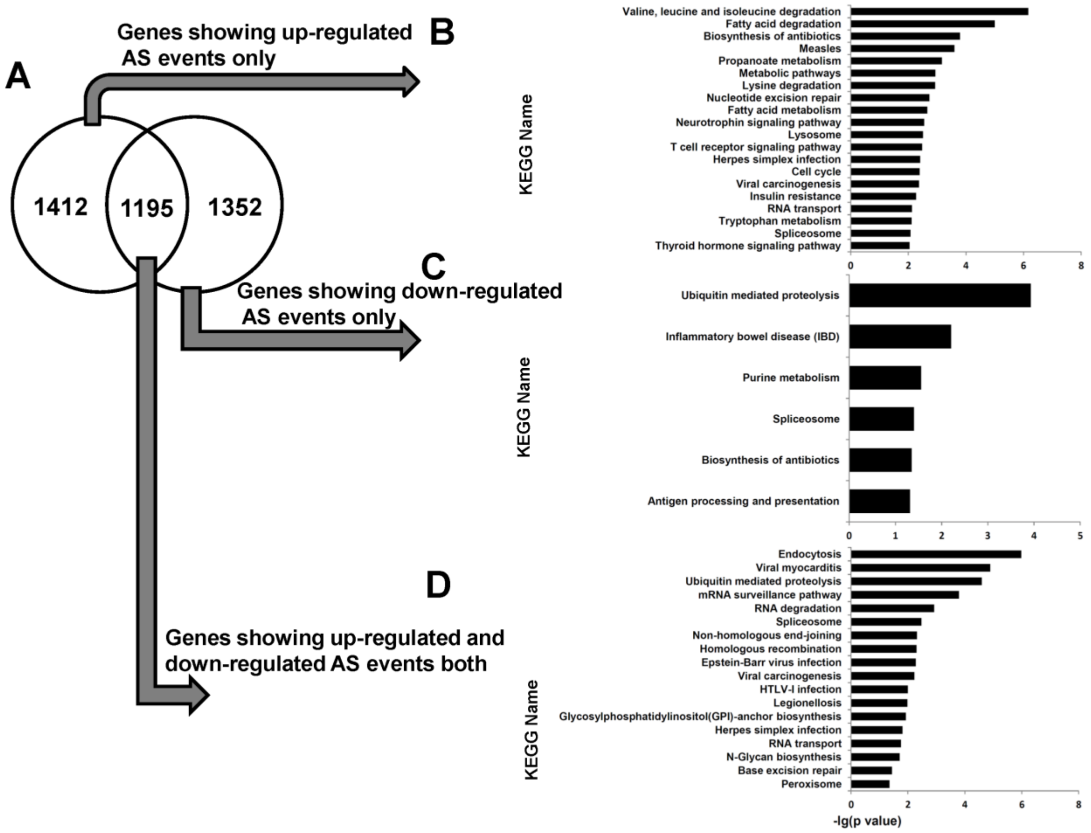

Figure S10. Venn diagram and KEGG pathway analysis for the genes undergoing upregulated and downregulated AS events in dCD4 T cells. (A-D) Venn diagram (**A**) and KEGG pathway analysis (**B-D**) for genes undergoing upregulated (FDR < 0.05 with $\Delta\Psi > 0.05$ between samples) and downregulated (FDR < 0.05 with $\Delta\Psi < -0.05$ between samples) AS events (SE, MXE, A5SS, A3SS and RI are combined together) in dCD4 T cells with respect to pCD4 T cells. KEGG, Kyoto Encyclopedia of Genes and Genomes; FDR, false discovery rate; SE, skipped exon; MXE, mutually exclusion exons; A5SS, alternative 5' splice site; A3SS, alternative 3' splice site; RI, retained intron.

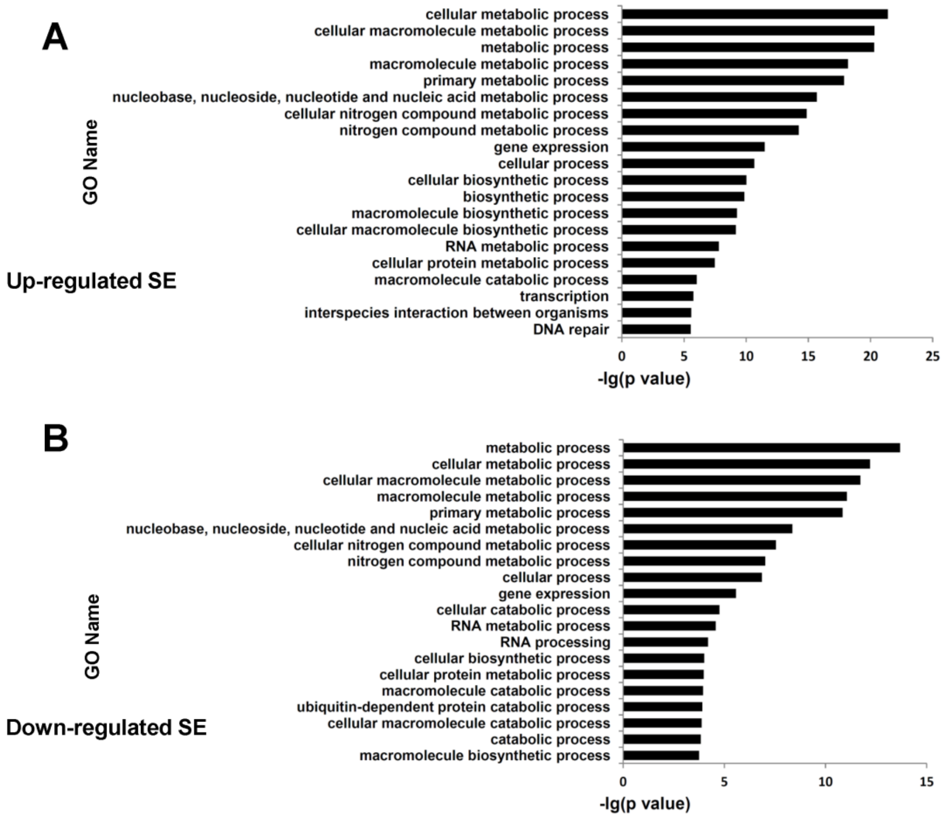

Figure S11. GO annotation for the genes undergoing upregulated or downregulated SE events in dCD4 T cells. (A and **B)** The top 20 GO terms enriched for genes undergoing upregulated (FDR < 0.05 with ΔΨ > 0.05 between samples; **A**) and downregulated (FDR < 0.05 with ΔΨ < -0.05 between samples; **B**) SE events in dCD4 T *versus* pCD4 T cells. GO, Gene Ontology; FDR, false discovery rate; SE, skipped exon.

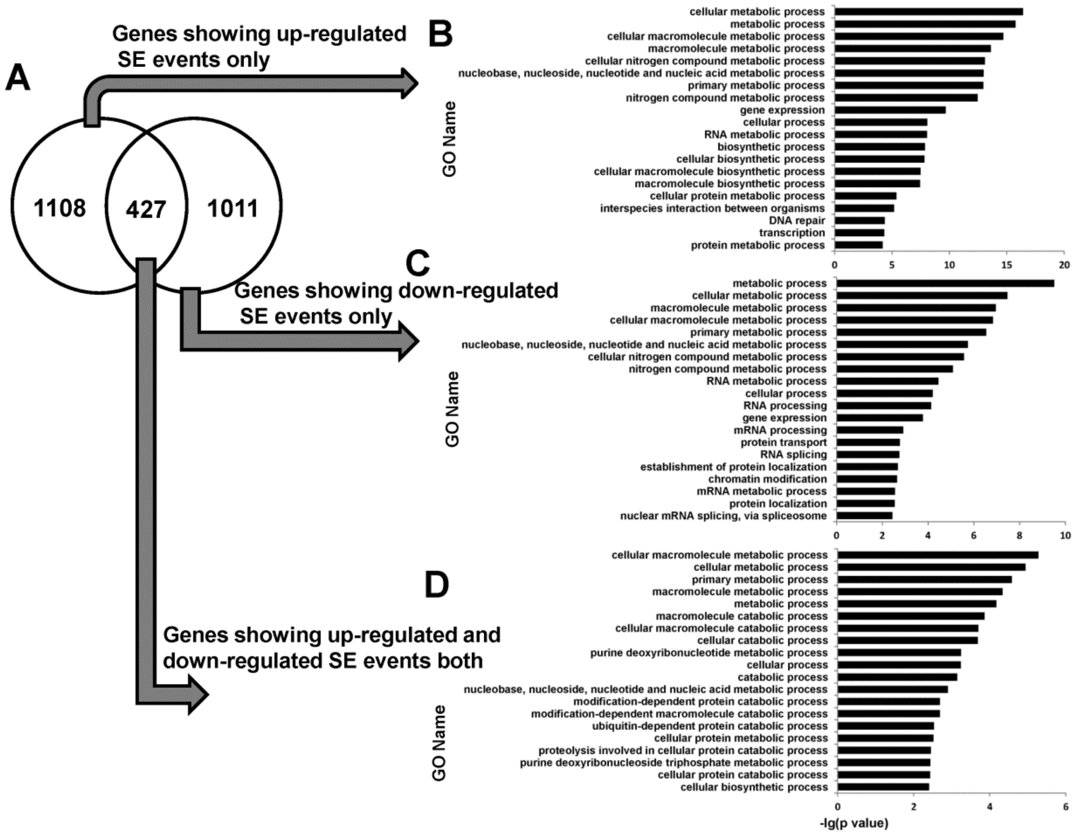

Figure S12. Venn diagram and GO annotation for the genes undergoing upregulated and downregulated SE events in dCD4 T cells. (A-D) Venn diagram (**A**) and GO annotation (**B**-**D**) for the genes undergoing upregulated (FDR < 0.05 with ΔΨ > 0.05 between samples) and downregulated (FDR < 0.05 with ΔΨ < -0.05 between samples) SE events in dCD4 T cells with respect to pCD4 T cells. GO, Gene Ontology; FDR, false discovery rate; SE, skipped exon.

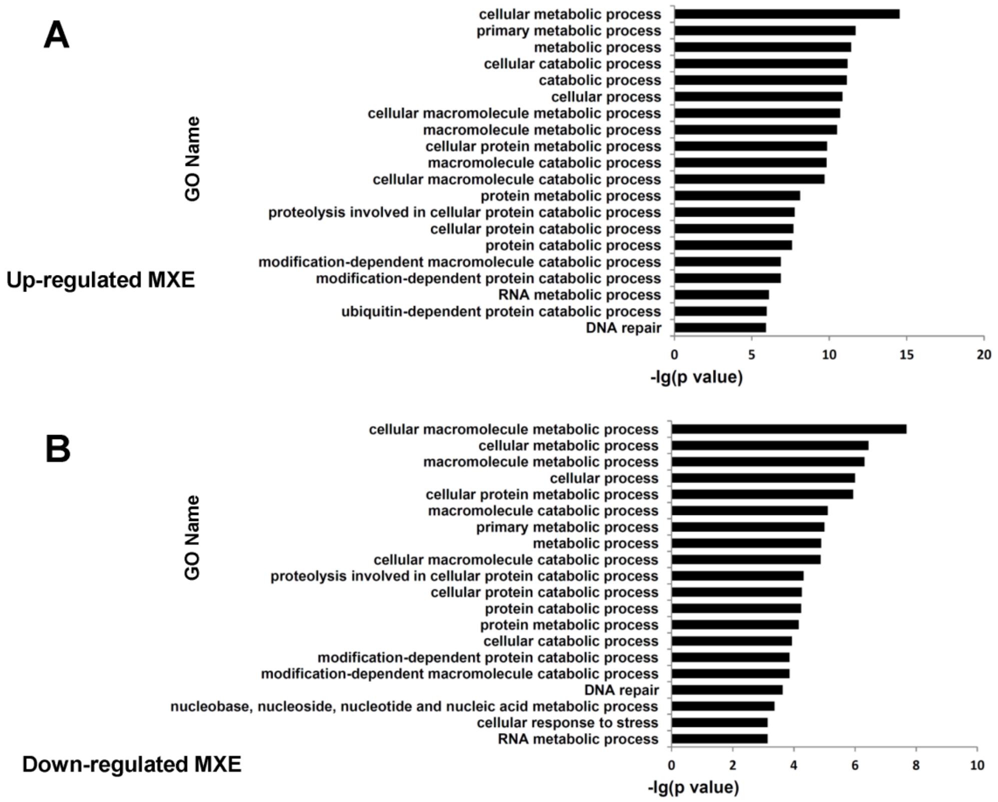

Figure S13. GO annotation for the genes undergoing upregulated or downregulated MXE events in dCD4 T cells. (A and **B)** The top 20 GO terms enriched for the genes undergoing upregulated (FDR < 0.05 with $\Delta\Psi > 0.05$ between samples; **A**) and downregulated (FDR < 0.05 with $\Delta\Psi < -0.05$ between samples; **B**) MXE events in dCD4 T *versus* pCD4 T cells. GO, Gene Ontology; FDR, false discovery rate; MXE, mutually exclusion exons.

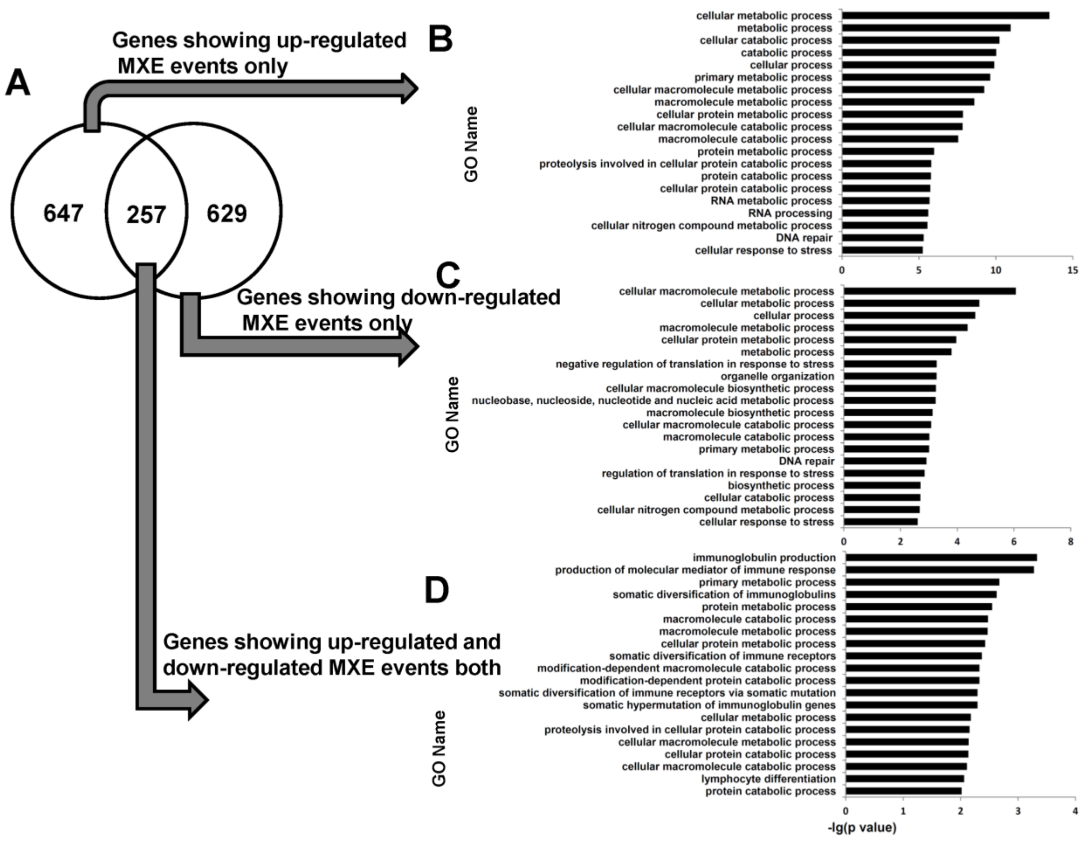

Figure S14. Venn diagram and GO annotation for the genes undergoing upregulated and downregulated MXE events in dCD4 T cells. (A-D) Venn diagram (**A**) and GO annotation (**B**-**D**) for genes undergoing upregulated (FDR < 0.05 with ΔΨ > 0.05 between samples) and downregulated (FDR < 0.05 with ΔΨ < -0.05 between samples) MXE events in dCD4 T cells with respect to pCD4 T cells. GO, Gene Ontology; FDR, false discovery rate; MXE, mutually exclusion exons.

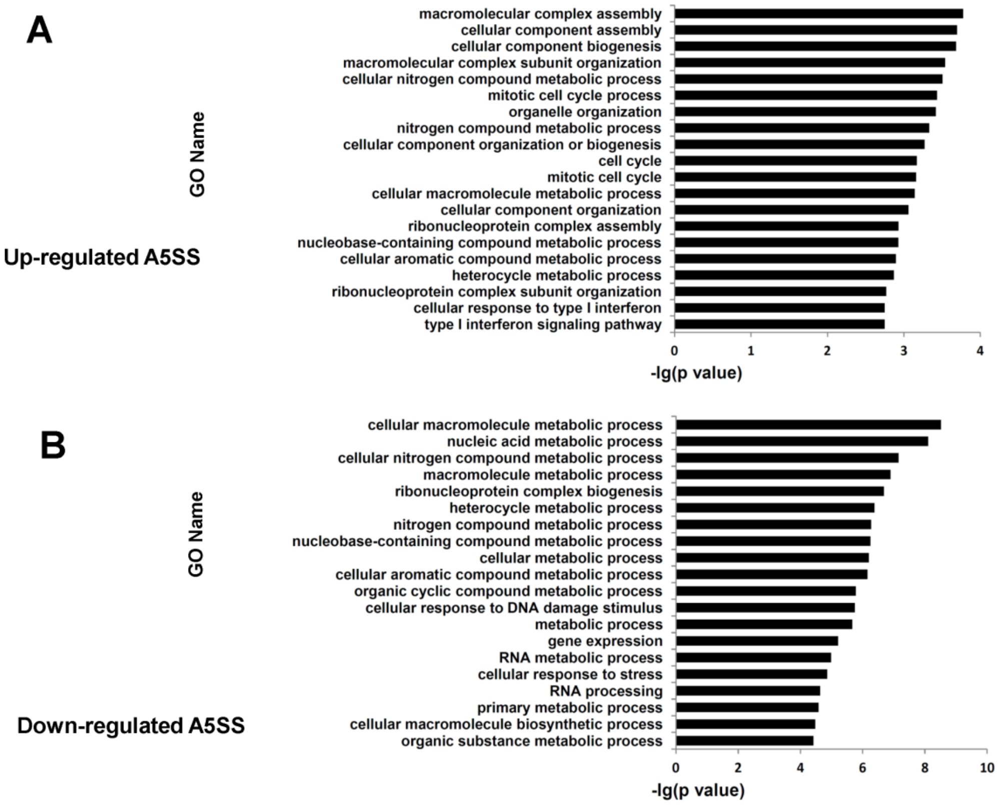

Figure S15. GO annotation for the genes undergoing upregulated or downregulated A5SS events in dCD4 T cells. (A and **B)** The top 20 GO terms enriched for genes undergoing upregulated (FDR < 0.05 with $\Delta\Psi > 0.05$ between samples; **A**) and downregulated (FDR < 0.05 with $\Delta\Psi < -0.05$ between samples; **B**) A5SS events in dCD4 T *versus* pCD4 T cells. GO, Gene Ontology; FDR, false discovery rate; A5SS, alternative 5' splice site.

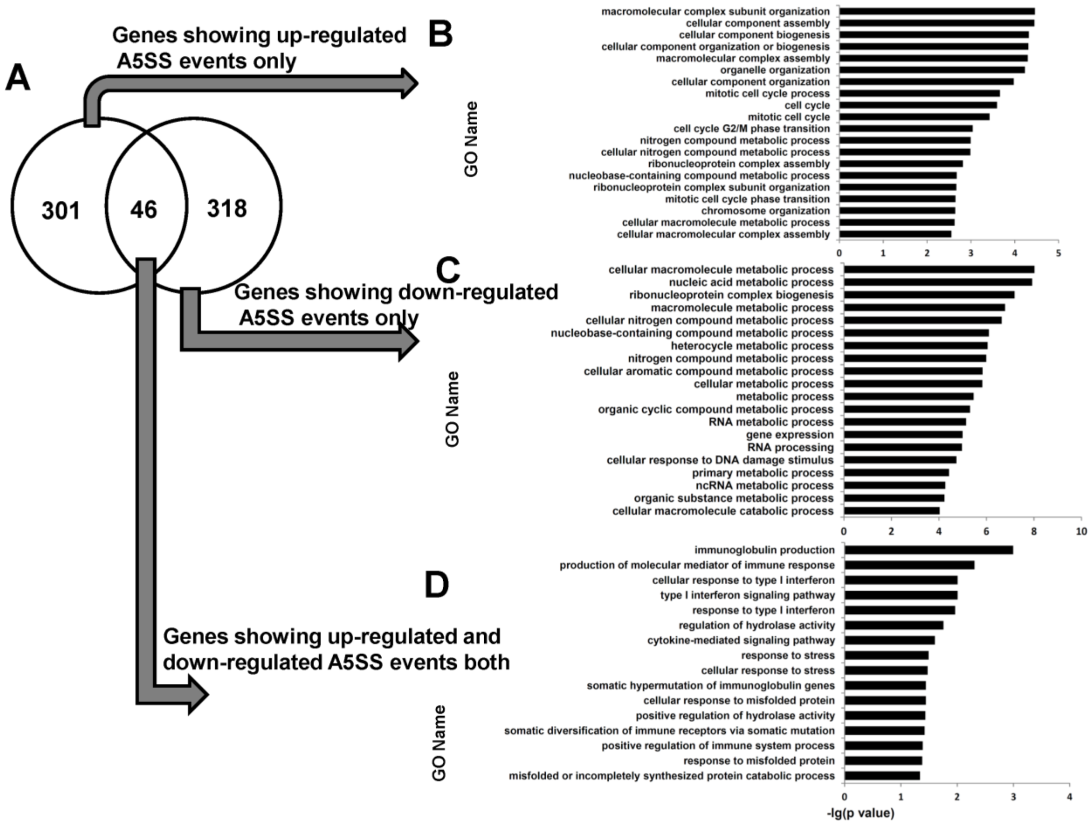

Figure S16. Venn diagram and GO annotation for the genes undergoing upregulated and downregulated A5SS events in dCD4 T cells. (A-D) Venn diagram (**A**) and GO annotation (**B-D**) for genes undergoing upregulated (FDR < 0.05 with ΔΨ > 0.05 between samples) and downregulated (FDR < 0.05 with ΔΨ < -0.05 between samples) A5SS events in dCD4 T cells with respect to pCD4 T cells. GO, Gene Ontology; FDR, false discovery rate; A5SS, alternative 5' splice site.

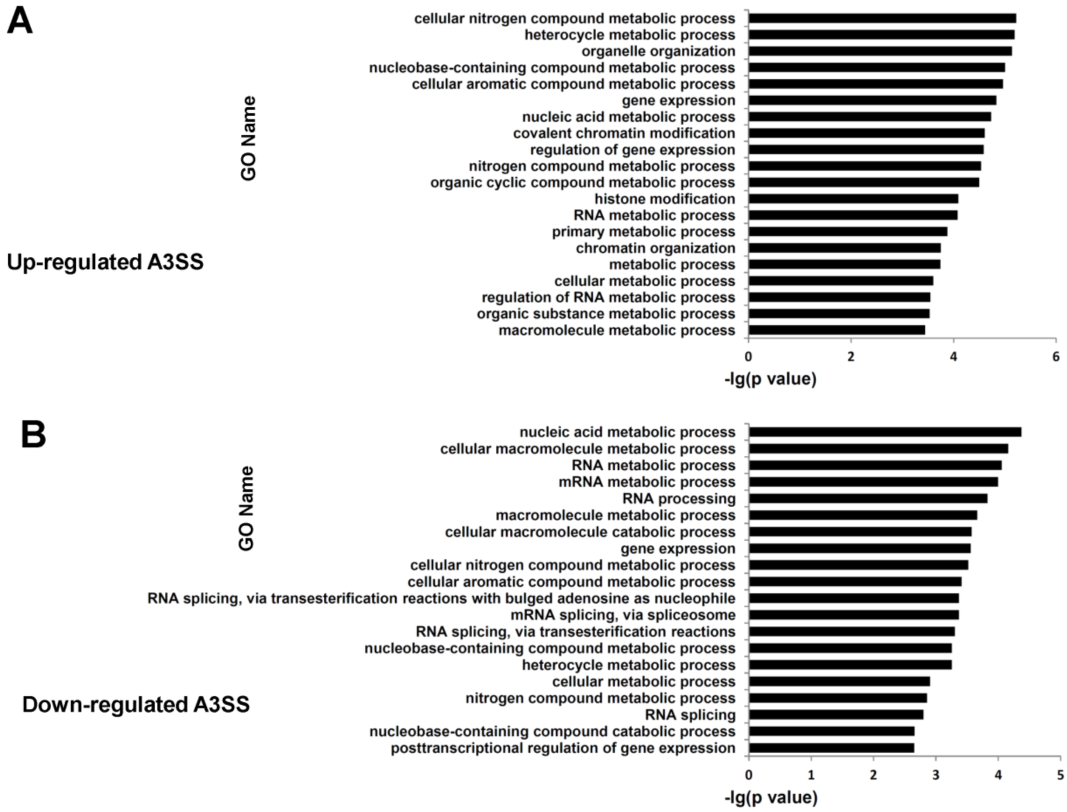

Figure S17. GO annotation for the genes undergoing upregulated or downregulated A3SS events in dCD4 T cells. (A and **B)** The top 20 GO terms enriched for genes undergoing upregulated (FDR < 0.05 with $\Delta\Psi > 0.05$ between samples; **A**) and downregulated (FDR < 0.05 with $\Delta\Psi < -0.05$ between samples; **B**) A3SS events in dCD4 T *versus* pCD4 T cells. GO, Gene Ontology; FDR, false discovery rate; A3SS, alternative 3' splice site.

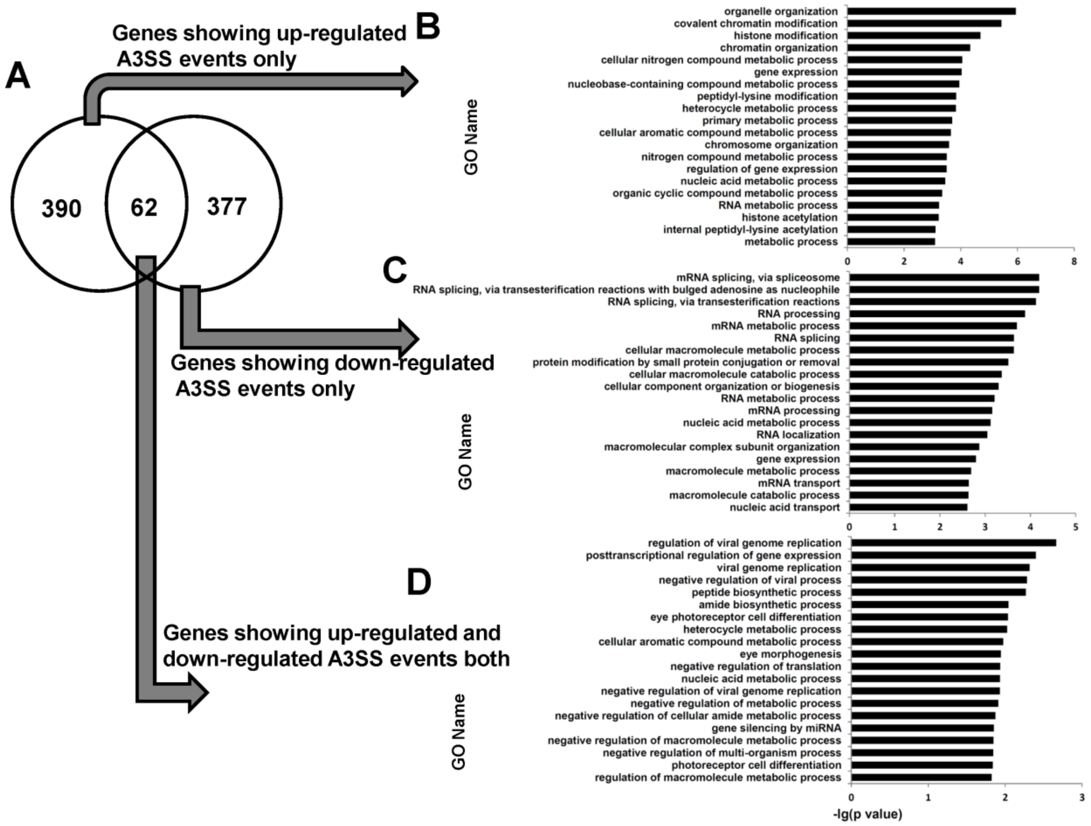

Figure S18. Venn diagram and GO annotation for the genes undergoing upregulated and downregulated A3SS events in dCD4 T cells. (A-D) Venn diagram (**A**) and GO annotation (**B-D**) for the genes undergoing upregulated (FDR < 0.05 with ΔΨ > 0.05 between samples) and downregulated (FDR < 0.05 with ΔΨ < -0.05 between samples) A3SS events in dCD4 T cells with respect to pCD4 T cells. GO, Gene Ontology; FDR, false discovery rate; A3SS, alternative 3' splice site.

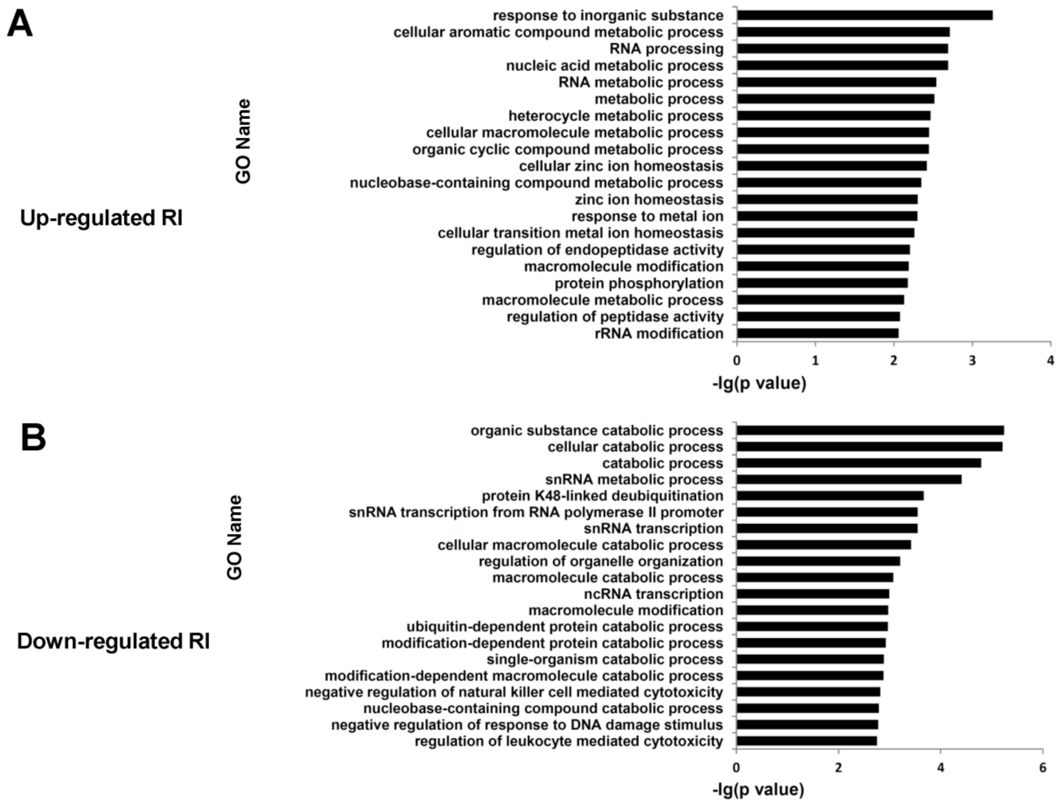

Figure S19. GO annotation for the genes undergoing upregulated or downregulated RI events in dCD4 T cells. (A and B) The top 20 GO terms enriched for genes undergoing upregulated (FDR < 0.05 with $\Delta\Psi > 0.05$ between samples; **A**) and downregulated (FDR < 0.05 with $\Delta\Psi < -0.05$ between samples; **B**) RI events in dCD4 T *versus* pCD4 T cells. GO, Gene Ontology; FDR, false discovery rate; RI, retained intron.

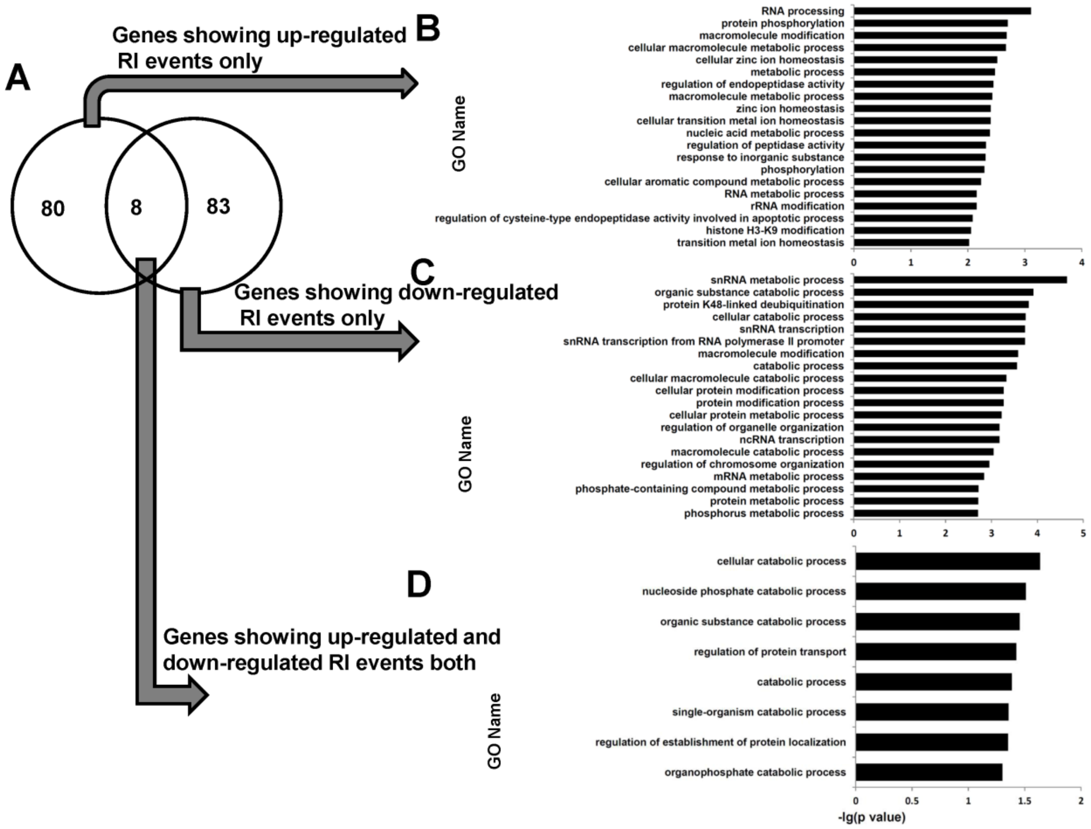

Figure S20. Venn diagram and GO annotation for the genes undergoing upregulated and downregulated RI events in dCD4 T cells. (A-D) Venn diagram (**A**) and GO annotation (**B-D**) for the genes undergoing upregulated (FDR < 0.05 with $\Delta\Psi > 0.05$ between samples) and downregulated (FDR < 0.05 with $\Delta\Psi < -0.05$ between samples) RI events in dCD4 T cells with respect to pCD4 T cells. GO, Gene Ontology; FDR, false discovery rate; RI, retained intron.

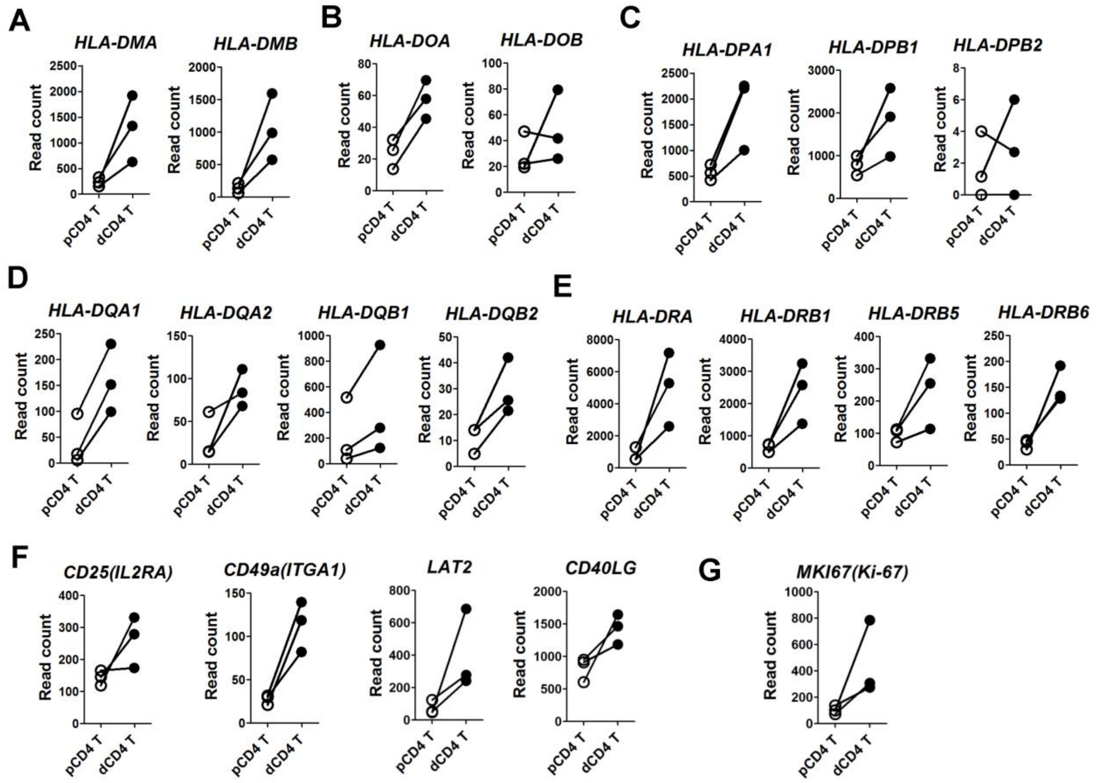

Figure S21. Comparison of the gene expression of MHC-II molecules (A-E), T-cell-activation antigens (F) and *MKI67* (G) between in paired pCD4 and dCD4 T cells. Gene expression quantification is measured as the read count. Each symbol reflects a sample and each line reflects the samples from the same individual(n = 3 per group).

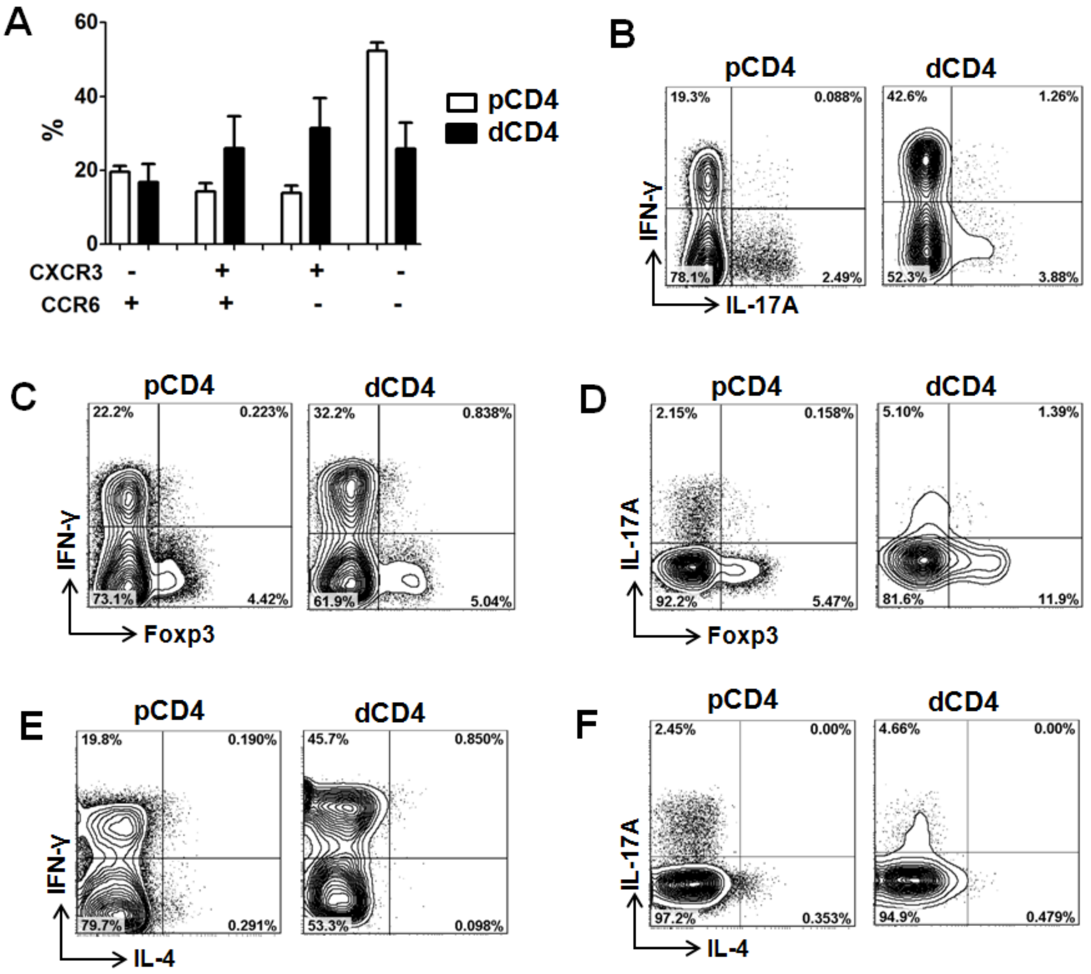

Figure S22. Co-expression analysis of indicated molecules in pCD4 and dCD4 T cells. (A) Co-expression of CXCR3/CCR6 in resting pCD4 and dCD4 T cells (n = 4). **(B-E)** Co-expression of IFN-γ/IL-17A **(B)**, IFN-γ/Foxp3 **(C)**, IL-17A/Foxp3 (D), IFN-γ/IL-4 **(E)** and IL-17A/IL-4 **(F)** in pCD4 and dCD4 T cells as determined by intracellular staining upon stimulation with PMA and ionomycin. Similar results were obtained from four individuals at the first trimester of normal pregnancy.

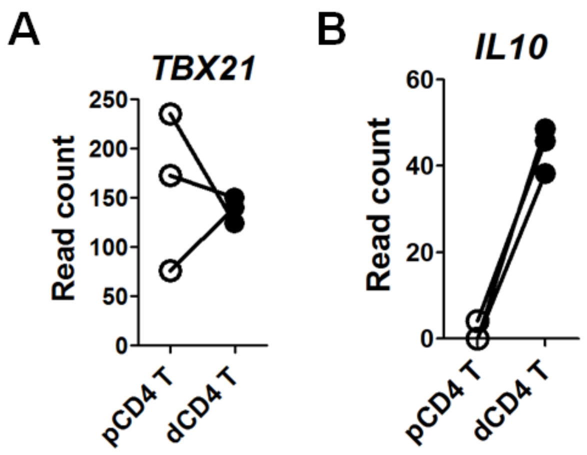

Figure S23. Comparison of *TBX21* and *IL10* expression between paired pCD4 and dCD4 T cells. Gene expression quantification is measured as the read count. Each symbol reflects a sample and each line reflects the samples from an individual (n = 3 per group).

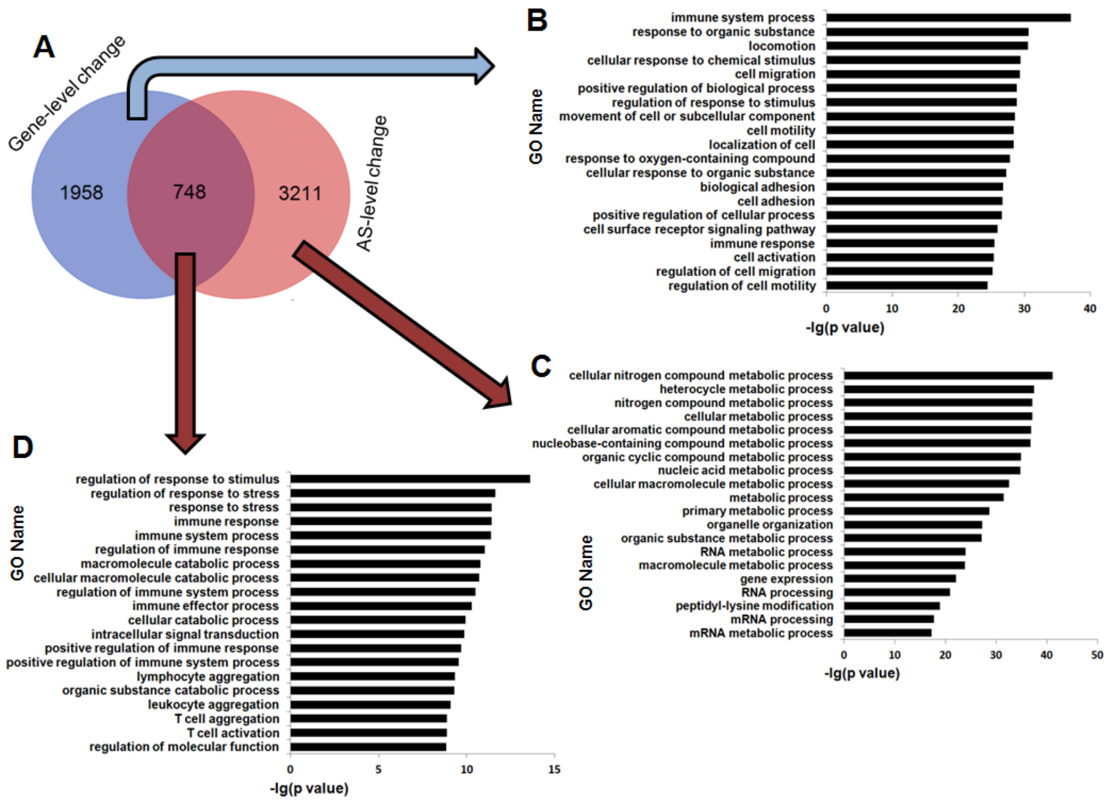

Figure S24. Venn diagram and GO annotation for the genes undergoing significant splicing-level and expression-level changes in dCD4 T cells. (A-D) Venn diagram (**A**) and GO annotation analysis (**B-D**) for genes undergoing significant expression-level ($p < 0.05$) and AS-level (FDR < 0.05 with $|\Delta\Psi| > 0.05$ between samples; SE, MXE, A5SS, A3SS and RI are combined together) changes in dCD4 T cells with respect to pCD4 T cells. GO, Gene Ontology; FDR, false discovery rate; SE, skipped exon; MXE, mutually exclusion exons; A5SS, alternative 5' splice site; A3SS, alternative 3' splice site; RI, retained intron.
